# Assessing the measurement properties of life-space mobility measures in community-dwelling older adults: a systematic review

**DOI:** 10.1093/ageing/afad119

**Published:** 2023-10-30

**Authors:** Ayse Kuspinar, Ava Mehdipour, Marla K Beauchamp, Qiukui Hao, Emily Cino, Christopher Mikton, Jotheeswaran Amuthavalli Thiyagarajan, Theresa Diaz, Parminder Raina

**Affiliations:** School of Rehabilitation Science, McMaster University, Hamilton, Ontario, Canada; McMaster Institute for Research on Aging, McMaster University, Hamilton, Ontario, Canada; School of Rehabilitation Science, McMaster University, Hamilton, Ontario, Canada; School of Rehabilitation Science, McMaster University, Hamilton, Ontario, Canada; McMaster Institute for Research on Aging, McMaster University, Hamilton, Ontario, Canada; School of Rehabilitation Science, McMaster University, Hamilton, Ontario, Canada; Faculty of Medicine, University of Ottawa, Ottawa, ON, Canada; Demographic Change and Healthy Aging Unit, Social Determinants of Health, World Health Organization, Geneva, Switzerland; Ageing and Health Unit, Department of Maternal, Newborn, Child and Adolescent Health and Ageing, WHO HQ, Geneva, Switzerland; Epidemiology, Monitoring and Evaluation Unit, Department of Maternal, Newborn, Child and Adolescent Health and Ageing, WHO HQ, Geneva, Switzerland; McMaster Institute for Research on Aging, McMaster University, Hamilton, Ontario, Canada; Department of Health Research Methods, Evidence, and Impact, McMaster University, Hamilton, Ontario, Canada; Labarge Centre for Mobility in Aging, McMaster, University, Hamilton, Ontario, Canada

**Keywords:** mobility, life-space, measurement properties, older people

## Abstract

**Background:**

Preserving and enhancing mobility is an important part of healthy ageing. Life-space mobility is a construct that captures actual mobility within the home and the community. The objective of this systematic review was to synthesise the measurement properties and interpretability of scores produced by life-space mobility measures in community-dwelling older adults.

**Methods:**

This systematic review followed Consensus-based Standards for the selection of health Measurement Instruments (COSMIN). Multiple databases were searched to identify potentially relevant articles. Data extraction and assessment of methodological quality was conducted by two independent reviewers. When possible, results were quantitatively pooled for each measurement property. If studies could not be combined quantitatively, then findings were summarised qualitatively using means and percentage of confirmed hypothesis. Synthesised results were assessed against the COSMIN criteria for good measurement properties.

**Results:**

A total of 21 full text articles were included in the review. The University of Alabama at Birmingham Study of Aging Life-Space Assessment (LSA) was the most evaluated life-space mobility measure. The LSA demonstrated content validity, internal consistency (Cronbach’s alpha 0.80–0.92), reliability [intra-class correlation value 0.89 (95% confidence interval (CI): 0.80, 0.94)] and convergent validity with measures of physical function in community-dwelling older adults.

**Conclusion:**

This systematic review summarised the measurement properties of life-space mobility measures in community-dwelling older adults following COSMIN guidelines. The LSA has been translated into multiple languages and has sufficient measurement properties for assessing life-space mobility among community-dwelling older adults.

## Key Points

- This systematic review synthesised the measurement properties of life-space mobility measures in community-dwelling older adults.
- The University of Alabama at Birmingham Study of Aging Life-Space Assessment (LSA) was the most evaluated life-space mobility measure.
- Scores produced by the LSA demonstrated evidence of reliability and validity in community-dwelling older adults.

## Introduction

The United Nations Decade of Healthy Ageing (2021–2030) calls for strengthening measurements for monitoring the healthy ageing and well-being of older people [1]. Preserving and enhancing mobility is an important outcome for healthy ageing policies, action plans, strategies and programmes [2]. Mobility, as described by the World Health Organization (WHO), is movement in all its forms, powered by the body or a vehicle [2]. Life-space mobility is a construct that refers to the movement of a person within their environment, extending from their home to the community and beyond [3–5]. A recent commentary presents a framework for comprehensive mobility measurement [6]. Within this framework, an older person’s ability to be mobile is made up of their perceived mobility ability (e.g. self-reported difficulty with walking) and their actual mobility performance in daily life (e.g. measures of counts or frequency of mobility activities). Life-space mobility measures make up the latter facet of mobility because they consider the spaces one visits in everyday life and the frequency of these visits over a specific time period [3]. These measures of actual or real-world mobility are gaining increased attention as an important indicator of healthy ageing.

Existing measures of life-space mobility include the Nursing Home Life-Space Diameter (NHLSD) [7], the Life Space Questionnaire (LSQ) [5] and the University of Alabama at Birmingham Study of Aging Life-Space Assessment (LSA) [8]. The NHLSD was developed for use in nursing homes, whereas the LSQ and LSA were developed for use in community-dwelling older adults. The LSQ asks about the life-space areas respondents visited over the past 3 days. The LSA asks about life-space areas visited during the past month, the frequency of these visits and if assistance was needed [8].

Although previous reviews have been published on life-space mobility measures, one was a narrative review [9] and the other focused on the use of these measures in the German language and included institutionalised settings [10]. The WHO is currently developing a measurement and evaluation framework for healthy ageing, which will include indicators for, and measurement of, functional ability. Within this framework, the ‘ability to be mobile’ is described as a key domain of functional ability—one of the three main components of healthy ageing. In another review [11], we summarised the psychometric properties of perceived measures of mobility ability. In this paper, we consider the psychometric properties of life-space mobility measures that reflect actual real-life mobility. The objective of this systematic review was to synthesise the measurement properties and interpretability of scores produced by life-space mobility measures, namely, the NHLSD, LSQ and LSA, in community-dwelling older adults.

## Methods

This systematic review followed COnsensus-based Standards for the selection of health Measurement INstruments (COSMIN) methodology for conducting systematic reviews of psychometric properties [12] and the Preferred Reporting Items for Systematic Reviews and Meta-Analyses (PRISMA) statement [13]. The protocol for this systematic review has been published [14].

### Criteria for considering studies for this review

Studies were included if they met the criteria listed below.

#### Population

Community-dwelling older adults aged 60 years of age or over. Studies were also included if the mean sample age was above 60 years or at least 50% of the sample was aged 60 years or over, or results were reported separately for persons aged 60 years or over. Older adults living in the community with chronic or neurological conditions were included.

#### Construct

The construct of interest was life-space mobility, which is an individual’s movement across environments starting from the home and extending to the world around them [3, 4, 5, 14].

#### Instruments

A preliminary search of life-space mobility measures and consultation with experts identified three main self-report measures that have been commonly used in older adults: the NHLSD, LSQ and LSA. These three measures and their modified versions were the focus of this review. The NHSLD was included if it had been adapted or used in community-dwelling older adults.

#### Outcomes

All psychometric properties were included [12]. Data on interpretability and feasibility were also extracted. Content validity of included measures was also evaluated [15].

#### Type of studies

Studies whose primary aim was to evaluate the measurement properties of life-space mobility measures were included in the review. Studies were not excluded based on design; all study designs were included. Grey literature and review articles were excluded.

#### Language

Only studies published in English were included.

### Search methods

MEDLINE (Ovid), Embase (Ovid) PsycINFO (Ovid) and CINAHL (EBSCO) were searched up to June 2021 with the terms ‘life-space’ or ‘life space’ to identify potentially relevant articles. The reference list of the included studies was also checked to ensure that any other potentially relevant studies were not missed.

### Data collection and analysis

#### Selection of studies

Two reviewers independently screened all the titles and abstracts. Studies that met the inclusion criteria were kept for full text-review. Two reviewers then independently reviewed the full-text articles and assessed their eligibility for inclusion. Those that met inclusion criteria were retained for further review.

#### Data extraction and management

Data extraction and assessment of methodological quality was conducted by two independent reviewers, and any disagreements between the reviewers were resolved by discussion with a third reviewer. The software Covidence [16] was used to import the references from the databases, screen titles and abstracts, and identify full text articles.

#### Assessment of content validity

The content validity of measures was evaluated following the steps outlined in the COSMIN manual for evaluating the content validity of patient-reported outcome measures [15].

#### Assessment of methodological quality

Assessment of methodological quality was performed using the COSMIN Risk of Bias (ROB) checklist (Box 3–10). Each measurement property from each study was rated as ‘very good’, ‘adequate’, ‘doubtful’ or ‘inadequate’. Furthermore, each measurement property from included studies was rated according to COSMIN’s criteria for good measurement properties (Appendix A). Each measurement property per study was rated as sufficient (+), insufficient (−) or indeterminate (?). When construct validity was assessed using correlation coefficient values, we hypothesised correlations ≥0.50 for measures capturing similar constructs; ≥0.30 for measures capturing related but distinct constructs; and <0.30 for unrelated constructs. For related but distinct constructs, although we expected a minimum correlation of 0.30, we did not specify an upper limit as the maximum correlation could vary. The direction of the correlations (+/−) was based on the underlying measurement scale of the instrument.

#### Statistical analysis and data synthesis

For content validity, individual ratings were summarised to determine an overall rating for content validity as sufficient (+), insufficient (−) or inconsistent (±). When possible, study results were pooled for each measurement property with forest plots. A random-effects model with transformation of coefficients was used for the analysis [17]. If considerable heterogeneity was present (*I*^2^ value > 75%), subgroup analysis using a random-effects model was conducted by study location (country), participant characteristics (average age, average number of comorbidities) and study quality (risk of bias). If studies could not be combined quantitatively, then findings were summarised qualitatively using means and percentage of confirmed hypothesis. All synthesised results were assessed against the COSMIN criteria for good measurement properties to determine a rating of sufficient (+), insufficient (−), inconsistent (±) or indeterminate (?). Results were sufficient if ≥75% of hypotheses were met and insufficient if ≥75% were not met. Inconsistent results were centred on ≥50% of ratings.

### Summary of the evidence and grading the evidence quality

COSMIN’s modified Grading of Recommendations Assessment and Development and Evaluation (GRADE) approach (Appendix B) was used to evaluate the quality of the pooled evidence per measurement property. Two independent reviewers graded the quality of the evidence, and any disagreements between the reviewers were resolved by discussion with a third reviewer. The evidence was graded as high, moderate, low or very low taking into consideration the following four factors: ROB, inconsistency, indirectness and imprecision [12]. For content validity studies, only ROB, inconsistency and indirectness were considered.

**Figure 1:**
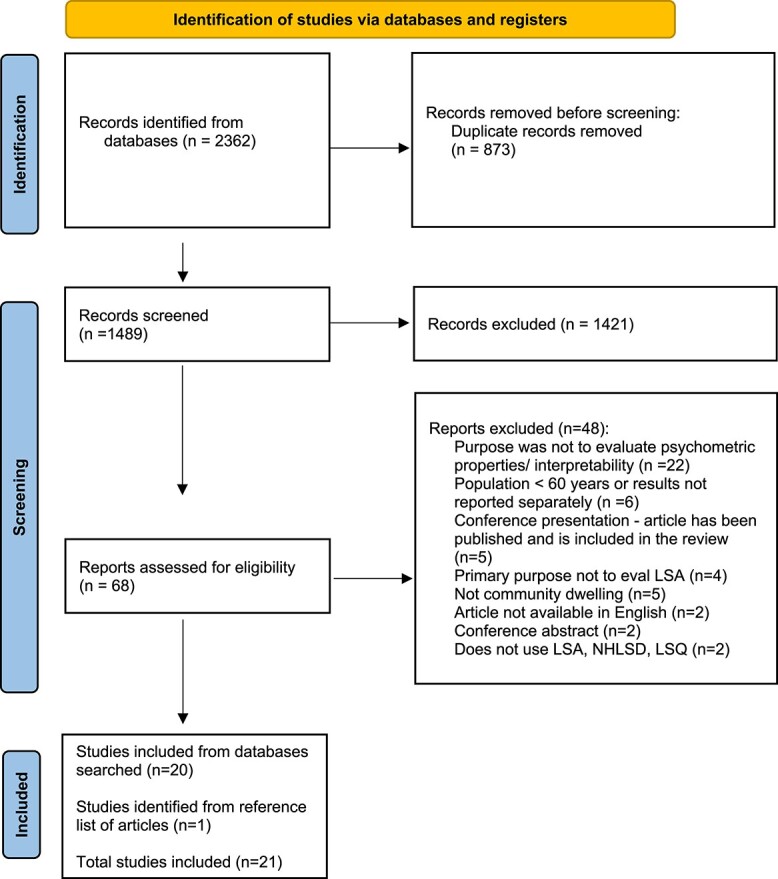
Flow diagram outlining study selection process [13].

## Results

### Study selection

From the four databases, 2,362 records were obtained and after removing duplicates, 1,489 records were screened. Sixty-eight full-text articles were reviewed, of which 48 were excluded (Figure 1). A total of 20 articles were included from the databases searched. Upon examination of the reference list for the included articles, an additional article was included, for a final total of 21 articles.

### Life-space mobility measures

Four measures emerged from the review: the LSA, a modified version of the LSA, the LSQ and a modified version of the LSQ. There were no studies that reported on the NHLSD in community-dwelling older adults. A description of the measures can be found in Appendix C. Seventeen out of the 21 articles evaluated the psychometric properties or the interpretability of the LSA composite score (LSA-C). Three articles assessed the modified LSA, one assessed the LSQ and one assessed the modified LSQ (Table 1).

**Table 1:** Study characteristics, interpretability and feasibility of the included life-space mobility measures

Author (year)	Measures assessed	Country	Sample size	Properties assessed	Mean Age (SD)	% Females	Population	Mean life space score	Change scores	Cut-off scores	Floor/ceiling effects	Completion time	Completion rate (for longitudinal studies)	Other interpretability findings
Ullrich *et al.* (2021)	Modified LSA-C; Modified LSA-E, LSA-I, LSA-M (German)	Germany	65 for convergent validity; 55 for reliability; 32 for responsiveness	Reliability; convergent validity; responsiveness; interpretability	81.4 ± 5.9	72.3	Older persons without cognitive impairment	Modified LSA-C scores ranged between 4.5 and 70			Ceiling effect for LSA-M with 35.4% of participants reaching the maximal; floor effect for LSA-I with 47.7% reaching the lowest possible score.	4.1 ± 2.1 minutes (range 1–10 minutes)	100% completion rate after 12-weeks	
Tseng *et al.* (2020)	LSA (Chinese)	Northern Taiwan	225 (40 for reliability); For content validity: 5 professionals	Reliability; convergent validity; content validity	72.60 (6.57)	70.70	65 or older; living in the community; normal cognitive function	69.74 (16.02)						
McCrone *et al.* (2019)	LSA	UK	276 for known groups; 273 for convergent validity; 228 for responsiveness	Convergent validity; known-groups validity; responsiveness	Median (range): 80.5 (25–99)	73.6	Community-dwelling, newly referred from outpatients; domiciliary, non-hospitalised; and domiciliary, recent hospital discharge	Median (range):20 (4–92)	Mean change between PT appointment and discharge appointment (avg: 8 weeks): 10.5 points (95% CI = 8.3–12.8)				82.6% had both assessment s completed (median time of 53 days)	
Ullrich *et al.* (2019)	Modified LSA-C; Modified LSA-E, LSA-I, LSA-M (german)	Germany	117 for convergent validity; 102 for reliability; 53 for responsiveness	Reliability; convergent validity; responsiveness; interpretability	82.3 (6.0)	76.3	Older persons with mild to moderate cognitive impairment discharged from geriatric rehabilitation	Modified LSA-C: 29.7 (15.4); LSA-E: 2.8 (1.4); LSA-I: 1.4 (1.6); LSA-M: 4.1 (1.1) (n = 102)			LSA-M: ceiling effect, with 29.9% obtaining the maximum score. LSA-I: floor effect with 49.6% obtaining the minimum score	Mean completion time (SD) to assess the Modified LSA-C was 4.1 (2.2) min.	100% completion rate after 12 weeks	
Ullrich *et al.* (2019)	Modified LSA-C; (German)	Germany	118 total sample; 108 for cut-off calculation	Interpretability	82.3 (6.0)	76.3	Older persons with mild to moderate cognitive impairment discharged home from geriatric rehabilitation	Modified LSA -C: 23.9 (13.2)		Modified LSA -C = 26.75 for differentiating people with low and high life-space mobility				
Kennedy *et al.* (2018)	LSA	USA	412	Interpretability	81.7 (4.77)	57.3	Adults 75 years and older participating in a longitudinal epidemiological study across the state of Alabama	64.6 (25.2)	MIC (improvement in walking status) = 2.5 points (95% CI = 1.3–3.8 points). MIC (decline) = 2.9 points (95% CI = 1.7–4.2 points). Effect size = 0.4; SRM = 1					
Garcia *et al.* (2018)	LSA (Brazilian version)	Brazil	62	Internal consistency; reliability; measurement error; convergent validity; interpretability	65 (range: 62–71)	39	Older adults with chronic obstructive pulmonary disease, in São Paulo, Brazil	Baseline: 62 (52–80)	90% MDC = 0.2 points					
Simoes *et al.* (2018)	LSA (Brazilian-Portuguese)	Brazil	80; For content validity: 10 professionals +30 participants	Internal consistency; reliability; measurement error; convergent validity; interpretability; content validity	70.04 (8.6)	61	Brazilian community-dwelling older adults age ≥ 60 years	52.86 (23.52)	90% MDC = 0.36 points					
Yang *et al.* (2017)	LSA (Korean)	Korea	34	Reliability; convergent validity	65.11(2.39)	41.18	Stroke patients receiving outpatient rehabilitation treatment	42.29 (3.68)						
Fristedt *et al.* (2016)	LSA-C, LSA-E, LSA-I, LSA-M	Sweden	312	Convergent validity	80 (5)	53	General older population in Sweden	LSA-C = 65 (23); LSA-E median and IQR = 5 (4–5); LSA-I median and IQR(Q1-Q3) = 4 (2–5); LSA-M median and IQR = 5 (5–5)						
Portegijs *et al.* (2016)	LSA	Finland	Baseline: 848; longitudinal: 755	Convergent validity; predictive validity; interpretability	Median: 80.4 (IQR 7.4)	62	75- to 90-year-old community-dwelling people living in Central Finland.	Median baseline score = 64 (IQR 30.4)	Decline >11.7 identified those who developed ADL inability over 2 years	LSA = 52.25 for identifying participants who developed ADL inability over 2 years				
Ji *et al.* (2015)	LSA (Chinese)	China	100 (40 for reliability)	Reliability; convergent validity	72.23 (5.05)	50	Older adults’ health records	71.87 (20.24)						
Cavanaugh *et al.* (2014)	LSA	USA	40	/	80.7 (7.5)	55	Community-dwelling older adult and his or her close companion	71.1 (95% CI = 61.8–80.3)						Self-report vs proxy version: ICC (1,1) = 0.88; 95% CI = 0.79–0.94; *P* < 0.001.)
Portegijs *et al.* (2014)	LSA-C, LSA-E, LSA-I, LSA-M (Finnish)	Finland	1-year: 808; Reliability: 39	Reliability; measurement error; responsiveness	80.6 (4.2)	/	Community-dwelling older people aged 75#90; living in central Finland	median (IQR) = 64 (3)			LSA-M: ceiling effects: 52%; LSA-I: ceiling effects: 44%; LSA-E: ceiling effects: 48%		95.28% at 1 year follow-up	
Curcio *et al.* (2013)	LSA-C, LSA-E, LSA-I, LSA-M (Spanish and Portuguese)	Colombia and Brazil	300 (39 for reliability)	Reliability; convergent validity	Colombia: 69.1 (6.4); Brazil: 69.6 (3.0);	50	Community-dwelling older adults (65–74 years of age)	Colombia: LSA-C = 51.85 (19.01); LSA-E = 3.96 (1.03); LSA-I = 3.80 (1.14); LSA-M = 4.16 (0.75) Brazil: LSA-C = 59.61 (17.75); LSA-E = 4.37 (0.82); LSA-I = 4.32 (0.91); LSA-M = 4.44 (0.69)						
Shimada *et al.* (2010)	LSA (Japanese)	Japan	Baseline: 2404; longitudinal: 436	Predictive validity; convergent validity; interpretability	Cross-sectional: 78.8(7.0); Longitudinal: 79.2(6.8)	Cross-sectional: 71.5; longitudinal: 72.5	All participants used preventive health care services provided by the Japanese long-term care insurance system.	Cross-sectional: 56.2 (28.1); longitudinal: 47.3 (20.1)		Cut points of IADLs limitation: 56 points using Youden index				
Auger *et al.* (2009)	LSA-C, LSA-E, LSA-I, LSA-M (French)	Canada	40	Reliability; content validity	65 (10)	57.5	≥ 50 who obtained a power motor device from a subsidised program	LSA-C = 25 (14); LSA-E = 1.8 (1.5); LSA-I = 0.2 (0.5); LSA-M = 3.8 (1.2)				Telephone version: 3–18 min (mean + SD: 9.2 + 3.9)		
Kammerlind *et al.* (2009)	LSA-C; LSA-E, LSA-I, LSA-M (Swedish)	Sweden	298	Reliability; measurement error; interpretability	80 (5)	51	Older persons in Sweden	LSA-C = 65.1 (22.4); LSA-E median (IQR) = 5.0 (4.0–5.0); LSA-I median (IQR): 4.5 (2.0–5.0); LSA-M median (IQR) = 5.0 (5.0–5.0)			LSA-C = 50% ceiling effect; LSA-E: 59% ceiling effect; LSA-I: 15% floor effect; LS-M: =79% ceiling effect			
Barnes *et al.* (2007)	LSQ -modified (0–6 levels)	USA	909	Convergent validity	80.89 (7.14)	74.6	Participants from retirement communities and subsidised housing facilities in and around the Chicago metropolitan area.	60.28% had a level 6 score (outside of town)						
Baker *et al.* (2003)	LSA-C, LSA-E, LSA-I, LSA-M	USA	306	Reliability; convergent validity	75.07(6.8)	54	Medicare beneficiaries aged 65 and older living in central Alabama	LSA-C: 62.9 (24.7); LSA-E: 4.1(1.1); LSA-I: 3.5(1.8); LSA-M: 4.6(0.7)	LSA-C showed at least a 10-point change for 50% of subjects (39% decreased and 11% increased) over 6 months					
Stalvey *et al.* (1999)	LSQ	USA	242 (200 for reliability)	Reliability; measurement error; convergent validity	27% age 55–64, 51% age 65–74 and 22% age 75–85	44	Older adults were recruited from eye care clinics in Alabama	6 (1.4)					83% had their 2^nd^ assessment completed (test-retest at 1-year)	

CI, confidence interval; LSA, Life-Space Assessment; LSA-C, Life-Space Assessment-Composite; LSA-E, Life-Space Assessment-Equipment; LSA-M, Life-Space Assessment-Maximal; LSA-I, Life-Space Assessment-Independent; LSQ, Life-Space Questionnaire; MID, minimal important change; SD, standard deviation.

### Study characteristics

The included studies had a large range in sample size, ranging from 32 [18] to 2,404 [19] participants (Table 1). The included studies were conducted in Canada [20], Sweden [21, 22], United States [5, 8, 23–25], Finland [26, 27], Brazil [28–30], Korea [31], Colombia [30], China [32], Japan [19], United Kingdom [33], Northern Taiwan [34] and Germany [18, 35, 36].

### Measurement properties

Table 2 outlines the summary of findings for each measurement property and its quality of evidence for the LSA-C. Summary of findings tables can be found in Appendix D for the LSA subscales, the LSQ and the modified versions of these measures. Appendix E gives an overview of GRADE for each property, for each measure. Results of each study and their ratings (ROB and criteria for good measurement properties) can be found in Appendix F.

**Table 2:** Summary of findings table for the LSA-composite

Property	# of tests/hypotheses	Summary or pooled result	Overall rating	Quality of evidence
Internal consistency	2	100% of hypotheses were confirmed; Cronbach alpha: 0.8–0.92	Sufficient	High
Reliability	10	100% of hypotheses were confirmed; ^a^see forest plot; overall ICC = 0.89 (0.80–0.94)	Sufficient	High
Measurement error	3	0% of hypotheses were confirmed; SEM ranges from 4.12 to 9.1	Insufficient	High
Content validity	3	86% of items of the French version corresponded to the original version; LSA explains its conceptual model and administration instructions, and no professional made considerations or had concerns about the concept of life-space mobility; the item-level content validity index (assess relevancy of items) was 1.00 and the scale level was 0.86 for the LSA-C scale	Sufficient	Moderate^a^
Predictive validity	2	100% of hypotheses were confirmed; able to identify those with declines in ADLs/IADLs	Sufficient	High
Convergent validity	36	78% of hypotheses were confirmed; ^a^see forest plots. Overall correlation with IADL: 0.53 (0.38–0.66); with GDS: −0.51 (−0.63- (−0.36))	Sufficient	High
Known-groups validity	3	67% of hypotheses were confirmed	Sufficient; inconsistent	Very low^b^
Responsiveness	4	50% of hypotheses were confirmed	Sufficient; inconsistent	Moderate^c^

ADL, Activities of Daily Living; IADL, Instrumental Activities of Daily Living; GDS, Geriatric Depression Scale; ICC, intraclass correlation coefficient; SEM, standard error of measurement.

^a^Only considers risk of bias, inconsistency and indirectness in GRADE. No issues with inconsistency and indirectness were found. There was at least one doubtful rating, therefore downgraded to moderate quality of evidence.

^b^Very serious risk of bias and serious inconsistency

^c^Serious inconsistency

### Content validity

Three studies reported on the content validity of the LSA.

Simoes *et al.* asked 10 professionals and 30 older adults about the questionnaire’s comprehensiveness, and they reported no concerns with the LSA reflecting its conceptual model, its administration instructions or the concept of life-space mobility [28]. Tseng *et al.* administered a 4-point item-level and scale-level content validity index to five professionals to assess relevancy and reported good content validity [34]. Auger *et al.* evaluated the content validity of the French version of the LSA and reported that 86% of items corresponded to the English version [20]. The relevance, comprehensiveness and comprehensibility of the LSA was met according to the reviewers and the three studies.

When examining the development of the LSA [3], relevance of the LSA was rated as sufficient, and comprehensiveness and comprehensibility were rated as insufficient as concept elicitation and cognitive interviewing were not performed. However, as content validation studies provide more current, robust evidence, overall ratings for the LSA were sufficient. Moderate quality of evidence was found as there were doubtful ratings for ROB, mainly due to a lack of information on how the studies were conducted.

The content validity of the LSQ was assessed by examining the development study by Stalvey *et al.* [5] and reviewer ratings. Although reviewers rated the relevance, comprehensiveness and comprehensibility of the LSQ as sufficient, Stalvey *et al.* did not report on concept elicitation or cognitive interviewing, causing comprehensiveness and comprehensibility to receive an insufficient rating. Therefore, the overall quality of evidence of the LSQ was rated as very low due to inconsistent ratings, a lack of content validation studies and indirectness from the population (i.e. population from single site; an eye care clinic).

A content validity analysis on the modified versions of the LSQ and LSA were performed using the papers on the development of the original versions, the development of the modified versions and reviewer ratings. Similar to the original versions, overall quality of evidence was moderate for the modified version of the LSA and very low for the modified version of the LSQ.

### Internal consistency

Two studies [28, 29] evaluated the internal consistency of the LSA and reported Cronbach’s alphas of 0.80 and 0.92. Both had very good ROB ratings and the overall quality of evidence was high (Table 2).

### Reliability

**Figure 2:**
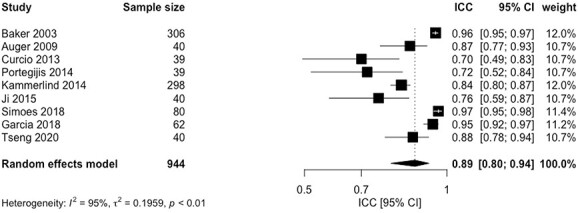
Forest plot intraclass correlation coefficient values.

Twelve studies [8, 18, 20, 22, 27–32, 34, 35] evaluated the test-retest reliability of the LSA or its modified version, and one study [5] evaluated the test-retest reliability of the LSQ (Appendix F). The second administration of the test occurred between 2 days and 2 weeks for the LSA and its subscales and 1 year for the LSQ. A pooled intraclass correlation coefficient (ICC) value of 0.89 (95% confidence interval (CI): 0.80, 0.94) was obtained for the LSA-C (Figure 2). The *I*^2^ value for the pooled results was 95%; therefore, subgroup analysis was performed by risk of bias and mean age. The sub-group analysis was statistically significant for risk of bias (*P* = 0.02) but not for mean age (*P* = 0.48). The forest plots for these analyses are presented in Appendix G. Sub-group analysis by country was not performed, as this variable could not be categorised into groups. Subgroup analysis by average number of comorbidities could also not be assessed because this information was not included in a number of studies.

For the LSA subscales, ICC values ranged from 0.37 to 0.86 for life-space with equipment (LSA-E), 0.63–0.94 for independent life-space (LSA-I) and 0.49–0.81 for maximum life-space (LSA-M). A weighted kappa of 0.80 was reported for the LSQ (Appendix F). The overall quality of evidence for the LSA-C score was high, whereas the LSA subscales had very low to low qualities of evidence with very serious ROBs (i.e. multiple studies of inadequate quality or one study of doubtful). The LSQ had very low quality of evidence as there was extremely serious risk of bias (i.e. only one study with inadequate risk of bias).

### Measurement error

Three studies evaluated the measurement error of the LSA-C, [22, 28, 29] two studies examined the LSA subscales, [22, 27] and one study examined the LSQ [5]. The standard error of measurement (SEM) for the LSA-C ranged from 4.12 to 9.1. These values were higher than the minimal important change (MIC) value [23]; therefore, hypotheses were not met (please refer to Appendix A for hypotheses). However, there was a high quality of evidence for this property. Percent agreement for LSA subscales ranged from 59 to 81%, with a low quality of evidence for the LSA-E and moderate for the LSA-I and LSA-M.

### Predictive validity

Two studies looked at the predictive validity of the LSA-C [19, 26]. Portegijis *et al.* evaluated whether baseline LSA-C scores could identify those who developed ADL difficulties at 2 years and reported an AUC of 0.79 [26]. Shimada *et al.* reported an odds ratio of 2.18 (95% CI: 1.26–3.79) and a c-index of 0.84 (*P* < 0.001) for the LSA-C predicting functional decline in Instrumental Activities of Daily Living (IADL) at 1 year [19]. These studies had a sufficient rating with an overall high quality of evidence (Table 2 and Appendix F).

### Convergent validity

Nine reports [8, 19, 21, 28, 29, 31–34] examined the convergent validity of the LSA-C, and two reports evaluated the modified LSA [18, 35]. One report evaluated the convergent validity of the LSQ [5] and another one the modified LSQ [25].

Forest plots were performed for LSA-C against measures of IADL and the Geriatric Depression Scale (GDS) (Appendix H). Pooled correlations were 0.53 (95% CI: 0.38–0.66) against IADL and −0.51 (95% CI: −0.63- (−0.36)) against GDS. For the LSA-C, correlations ranged from 0.37 to 0.60 with performance-based measures and 0.43 to 0.63 with accelerometry data. For LSA-C, 78% of the 36 hypotheses (Appendix A) were confirmed, with a high quality of evidence (Table 2). With performance-based measures, correlations were 0.47 for the LSA-E, 0.60–0.63 for the LSA-I and 0.19–0.20 for the LSA-M. The LSQ had moderate quality of evidence, with 25% of hypotheses confirmed.

### Known-groups validity

One report [33] evaluated the known-groups validity of the LSA-C testing three hypotheses. McCrone *et al.* evaluated the mean difference between three cohorts: (i) outpatients (ii) domiciliary, non-hospitalised and (iii) domiciliary, recent hospital discharge. We expected at least a 3-point difference between the cohorts [23] and that this difference was statistically significant. McCrone *et al.* reported a statically significant difference of 14.1 between cohorts 1 and 2 (*P* < 0.001), a statistically significant difference of 17.3 between cohorts 1 and 3 (*P* < 0.001) and a difference of 3.3 with an unreported *P*-value between cohorts 2 and 3 [33]. The overall quality of evidence was very low due to very serious ROB because of poor reporting, inconsistent ratings and very serious indirectness due to having an age range of 25–99 years (Table 2).

### Responsiveness

Four studies [18, 27, 33, 35] evaluated responsiveness. For LSA-C, 50% of hypotheses were confirmed [27, 33] and a standardised response mean (SRM) value of 0.60 was reported with physiotherapy intervention [33]. Portegijis *et al.* evaluated the correlation between LSA scores and perceived change in mobility over 1 year and reported a correlation of 0.22; however, we expected the correlation to be at least 0.30 [27]. The overall quality of evidence for the LSA-C for this property was moderate due to inconsistent ratings.

Two studies reported SRM values 0.70 and 0.80 following a home-based exercise program for the modified LSA-C [18, 35]. These two studies [18, 35] also evaluated the responsiveness of the subscales of the modified LSA. Hypotheses were 100% met for the subscales of the modified LSA, with SRMs from rehabilitation interventions ranging from 0.33 to 0.35 for LSA-E, 0.43 to 0.46 for LSA-I and 0.48 to 0.60 for LSA-M. The overall quality of evidence was moderate for the subscales due to Imprecision (i.e. samples less than 100) and serious ROB from doubtful ratings resulting from no predefined expectations of SRM magnitudes.

### Feasibility and interpretability

Mean completion time for the LSA was 9.2 minutes over the phone [20]. For the modified LSA, the mean completion time was reported as 4.1 minutes when answered in person [18, 35]. Longitudinal studies reported a completion rate of 95.28% for the LSA [27] and 83% for the LSQ [5], at 1 year (Table 1).

Kennedy *et al.* evaluated MIC values for the LSA-C using a walking status anchor at monthly intervals [23]. An MIC of 2.5 points was reported for improvements in walking status, and an MIC of 2.9 points was reported for declines in walking status. Portegijis *et al.* reported a decline of >11.7 points on the LSA-C for those who developed ADL inability over 2 years [26]. Baker *et al.* reported at least a 10-point change in LSA over 6 months in 50% of participants [8], and McCrone *et al.* reported a mean change of 10.5 points between physiotherapy initial assessment and discharge, over an average of 8 weeks [33]. LSA-C cut-points for detecting ADL/IADL difficulties were 52.25 and 56 [19, 26]. A ceiling effect of 50% was reported for the LSA-C, [22] 48–59% reported for the LSA-E, [22, 27] 44% reported for the LSA-I, [27] and 29.9–79% reported for the LSA-M [18, 22, 27, 35]. For the modified LSA, a cut-off value of 26.75 differentiated between people with low and high life-space mobility [36]. Results related to interpretability are presented in Table 1.

One study assessed agreement between the LSA when completed by the participant versus their companion, and it reported an ICC of 0.88 between the two versions [24].

## Discussion

This systematic review synthesised the measurement properties of life-space mobility measures in community-dwelling older adults following COSMIN guidelines. A total of 21 studies emerged from this review, of which the majority evaluated the University of Alabama at Birmingham Study of Aging LSA. The LSA assesses the actual day-to-day extent of movement across five different life-space areas, the frequency of this movement and whether assistance is needed or not. The LSA has been translated into multiple languages and has demonstrated content validity in community-dwelling older adults. This review identified that there is evidence in support of its reliability and validity in community-dwelling older adults.

The life-space mobility measures included in this review were developed based on the concept of life-space, which is broadly defined as an area that an individual moves through in daily life within a specific time frame [3]. Although performance-based tests such as the Time-Up-and-Go test or the gait speed test can be useful for evaluating one’s locomotor capacity for mobility, they are often performed in standardised settings that may not always be reflective of how one functions in daily life. Similarly, perceived measures of mobility ability do not always reflect actual day-to-day movement [37]. Life-space mobility measures provide an opportunity to assess actual or enacted mobility within the home or community setting and can complement measures of perceived mobility or capacity.

Responsiveness is an important psychometric property, especially when an outcome measure is used to evaluate treatment effects or change over time. This review identified four studies that evaluated the responsiveness of the LSA: two for the original version [27, 33] and two for the modified version [18, 35]. Among the included studies, the SRM values ranged from 0.60 to 0.80 (moderate to large) for the LSA or its modified version. However, distribution-based methods like the SRM can be affected by standard deviation values, the sample under study and variations in the treatment effect [38]. Therefore, future responsiveness studies evaluating change in scores on the LSA with change in scores on other mobility measures that have demonstrated responsiveness in community-dwelling older adults, are needed.

This review had several strengths. We closely followed COSMIN’s internationally accepted guidelines for systematically evaluating the measurement properties of patient-reported outcomes [12]. Furthermore, we evaluated the quality of the pooled evidence per measurement property using the modified GRADE approach. Also, as per COSMIN recommendations, we rated the measurement properties and methodological quality of the composite scores separately from the subscale scores, as the overall performance of each one might be different.

A limitation of this review was that we only included articles published in English, therefore, studies that evaluated the measurement properties of life-space mobility measures in other languages may have been missed. Moreover, we only included studies whose objectives were to evaluate the measurement properties of life-space mobility questionnaires. Articles that reported associations between life-space mobility and other variables, but were not measurement studies were excluded because they would have been assessed unfairly for quality. Therefore, we might have missed studies that could be informative, but did not meet the criteria for inclusion. In addition, when creating hypotheses against related but distinct constructs (e.g. GDS), a maximum correlation was not provided which could affect overall ratings. Furthermore, the instruments were usually tested in middle- or high-income countries or urban settings, which may limit the generalisability of our results. There were also deviations from the original protocol that should be noted. To align with COSMIN recommendations, grey literature, editorials and conference proceedings were excluded, as these often provide limited information and can impede quality assessment. Furthermore, as publication bias and selective reporting bias is difficult to quantify with studies on measurement properties, these were not assessed. Also, the eligibility criteria for age was reduced from 65 years old to 60, to better reflect the definition of an older person from the WHO.

In conclusion, based on our findings and synthesis of the literature, the LSA has sufficient measurement properties for assessing life-space mobility of older persons living in the community. There is a need for further well-designed studies on the responsiveness of the LSA in community-dwelling older adults and on its modified versions in general.

## Supplementary Material

aa-23-0362-File002_afad119

aa-23-0362-File003_afad119

aa-23-0362-File004_afad119

aa-23-0362-File005_afad119

aa-23-0362-File006_afad119

aa-23-0362-File007_afad119

aa-23-0362-File008_afad119

aa-23-0362-File009_afad119

## Data Availability

The data underlying this article are available in the article and in its online supplementary material.
