# Supplementary material for: Assessing the measurement properties of life-space mobility measures in community-dwelling older adults: a systematic review
Source: Age Ageing. 2023 Oct 30;52(Suppl 4):iv86–99. doi: 10.1093/ageing/afad119 (PMC10615067; doi:10.1093/ageing/afad119)
Supplement: aa-23-0362-File002_afad119 [file aa-23-0362-file002_afad119.docx]

**Appendix B. Modified GRADE approach for grading the quality of evidence**

| Quality of evidence | Lower if |
| --- | --- |
| High | **Risk of bias**  0 None: There are multiple studies of at least adequate quality, or there is at least one study of very good quality available.  -1 Serious: There are multiple studies of doubtful quality, or there is only one study of adequate quality available.  -2 Very serious: There are multiple studies of inadequate quality or there is only one study of doubtful quality available.  -3 Extremely serious: There is only one study of inadequate quality available.  **Inconsistency**  For inconsistent ratings only:  -1 Serious: If ≥50% of results were rated as sufficient according to COSMIN’s criteria for good psychometric properties)  -2 Very serious: If <50% of results were rated as sufficient according to COSMIN’s criteria for good psychometric properties    **Imprecision**  -1 Serious: If total (pooled/summarized) sample size is between 50 and 100.  -2 Very serious: If total (pooled/summarized) sample size is less than 50.    **Indirectness**  -1 Serious: At least 50% of studies had lower age limit within 10 years of 60 (i.e., 50) or 25% to 74% of study sample included older adults from retirement communities.  -2 Very serious: At least 50% of studies had lower age limits beyond 10 years of 60 (i.e., <50) or if ≥75% of study sample included older adults from retirement communities. |
| Moderate |  |
| Low |  |
| Very Low |  |
