# Supplementary material for: Assessing the measurement properties of life-space mobility measures in community-dwelling older adults: a systematic review
Source: Age Ageing. 2023 Oct 30;52(Suppl 4):iv86–99. doi: 10.1093/ageing/afad119 (PMC10615067; doi:10.1093/ageing/afad119)
Supplement: aa-23-0362-File003_afad119 [file aa-23-0362-file003_afad119.docx]

**Appendix A.**

**Table. Criteria for good measurement properties**

| Psychometric Property | Rating | Criteria |
| --- | --- | --- |
| Internal Consistency | + | Cronbach’s alpha(s) ≥ 0.70 for each unidimensional scale or subscale |
|  | ? | Cronbach’s alpha not reported |
|  | - | Cronbach’s alpha(s) < 0.70 for each unidimensional scale or subscale |
| Reliability | + | ICC, weighted Kappa, or correlations ≥0.70 |
|  | ? | ICC, weighted Kappa, or correlations not reported |
|  | - | ICC, weighted Kappa, or correlation <0.70 |
| Measurement Error | + | SDC or LoA <MIC |
|  | ? | MIC not defined |
|  | - | SDC or LoA >MIC |
| Hypothesis for Construct Validity | + | The result is in accordance with the hypothesis |
|  | ? | No statistics reported* |
|  | - | Results not in accordance with the hypothesis |
| Criterion Validity | + | Correlation with gold standard ≥ 0.70 OR AUC ≥ 0.70 |
|  | ? | No statistics reported |
|  | - | Correlation with gold standard < 0.70 OR AUC < 0.70 |
| Responsiveness | + | The result is in accordance with the hypothesis OR AUC ≥ 0.70 |
|  | ? | No statistics reported* |
|  | - | Results not in accordance with the hypothesis OR AUC < 0.70 |

The criteria are adapted and modified from COSMIN manual for systematic reviews [1] and based on Terwee et al. [2] and Prinsen et al. [3]

AUC = area under the curve, ICC = intraclass correlation coefficient, IRT = item response theory, LoA = limits of agreement, MIC = minimal important change, SDC = smallest detectable change

*This criteria has been modified from the COSMIN criteria for good measurement properties as the review team defined all hypotheses

1. Mokkink, Lidwine B., et al. "COSMIN methodology for systematic reviews of patient-reported outcome measures (PROMs)." User manual 78.1 (2018): 6-63.

2. Terwee, Caroline B., et al. "Quality criteria were proposed for measurement properties of health status questionnaires." Journal of clinical epidemiology 60.1 (2007): 34-42.

3. Prinsen, Cecilia AC, et al. "How to select outcome measurement instruments for outcomes included in a “Core Outcome Set”–a practical guideline." Trials 17.1 (2016): 1-10.

**Table. Hypotheses for criteria against good measurement properties**

| **Measure** | **Language** | **Author (year)** | **Internal consistency** | **Reliability** | **Measurement error** | **Predictive validity** | **Convergent validity** | **Known-groups validity** | **Responsiveness** |
| --- | --- | --- | --- | --- | --- | --- | --- | --- | --- |
| Modified LSA-C | German | Ullrich (2021) | / | We expect an ICC ≥ 0.70 within 3 days. | / | / | We expect a positive correlation of at least 0.5 with SPPB. We expect a negative correlation of at least 0.3 with FFABQ. We expect a positive correlation of at least 0.3 with walking episodes, steps. We expect a positive correlation of at least 0.5 with mean outdoor walking duration, mean outdoor walking distance, outdoor waling episodes. |  | We expect a SRM of at least 0.5 (based on Cohen’s d). |
| Modified LSA-E | German | Ullrich (2021) | / | We expect an ICC ≥ 0.70 within 3 days. |  |  | We expect a positive correlation of at least 0.5 with SPPB. We expect a negative correlation of at least 0.3 with FFABQ. We expect a positive correlation of at least 0.3 with walking episodes, steps. We expect a positive correlation of at least 0.5 with mean outdoor walking duration, mean outdoor walking distance, outdoor waling episodes. |  | We expect a SRM of at least 0.2 (based on Cohen’s d). |
| Modified LSA-I | German | Ullrich (2021) | / | We expect an ICC ≥ 0.70 within 3 days. |  |  | We expect a positive correlation of at least 0.5 with SPPB. We expect a negative correlation of at least 0.3 with FFABQ. We expect a pos correlation of at least 0.3 with walking episodes, steps. We expect a positive correlation of at least 0.5 with mean outdoor walking duration, mean outdoor walking distance, outdoor waling episodes. |  | We expect a SRM of at least 0.2 (based on Cohen’s d). |
| Modified LSA-M | German | Ullrich (2021) | / | We expect an ICC ≥ 0.70 within 3 days. |  |  | We expect a positive correlation of at least 0.5 with SPPB. We expect a negative correlation of at least 0.3 with FFABQ. We expect a pos correlation of at least 0.3 with walking episodes, steps. We expect a positive correlation of at least 0.5 with mean outdoor walking duration, mean outdoor walking distance, outdoor waling episodes. |  | We expect a SRM of at least 0.2 (based on Cohen’s d). |
| LSA | Chinese | Tseng (2020) | / | We expect an ICC ≥ 0.70 over 2 weeks. | / | / | We expect a positive correlation of at least 0.3 with PADL, CESD, SF-36 (GH), and 0.5 with IADL and MFAQ. |  |  |
| LSA | English | McCrone (2019) | / | / | / | / | We expect a positive a correlation of at least 0.5 with the POMA. | We expect a difference of at least 3 points between the cohorts and that this difference is statistically significant (p<0.05). | We expect a positive correlation of at least 0.3 between LSA and POMA differences. We expect a SRM of at least 0.5 (based on Cohen’s d). |
| Modified LSA-C | German | Ullrich (2019) | / | We expect an ICC ≥ 0.70 over 2 days. | / | / | We expect a positive correlation of at least 0.3 with SPPB, Gait speed and TUG. We expect a positive correlation of at least 0.2 with MMSE. We expect a negative correlation of at least 0.3 with GDS, FES-I, FFABQ. We expect a negative correlation of at least 0.3 with lying and sitting min and a positive correlation of at least 0.3 with standing, walking, walking episodes, steps. We expect a positive correlation of at least 0.5 with being active outdoors, mean outdoor walking duration, mean outdoor walking distance, outdoor waling episodes and max distance from home. |  | We expect a SRM of at least 0.5 (based on Cohen’s d). |
| Modified LSA-E | German | Ullrich (2019) | / | We expect an ICC ≥ 0.70 over 2 days. | / | / | We expect a positive correlation of at least 0.3 with SPPB, Gait speed and TUG. We expect a pos correlation of at least 0.2 with MMSE. We expect a negative correlation of at least 0.2 with GDS, FES-I, FFABQ. We expect a negative correlation of at least 0.3 with lying and sitting min and a pos correlation of at least 0.3 with standing, walking, walking episodes, steps. We expect a positive correlation of at least 0.5 with being active outdoors, mean outdoor walking duration, mean outdoor walking distance, outdoor waling episodes and max distance from home. |  | We expect a SRM of at least 0.2 (based on Cohen’s d). |
| Modified LSA-I | German | Ullrich (2019) | / | We expect an ICC ≥ 0.70 over 2 days. | / | / | We expect a positive correlation of at least 0.3 with SPPB, Gait speed and TUG. We expect a pos correlation of at least 0.2 with MMSE. We expect a negative correlation of at least 0.3 with GDS, FES-I, FFABQ. We expect a negative correlation of at least 0.3 with lying and sitting min and a pos correlation of at least 0.3 with standing, walking, walking episodes, steps. We expect a positive correlation of at least 0.5 with being active outdoors, mean outdoor walking duration, mean outdoor walking distance, outdoor waling episodes and max distance from home. |  | We expect a SRM of at least 0.2 (based on Cohen’s d). |
| Modified LSA-M | German | Ulrich (2019) | / | We expect an ICC ≥ 0.70 over 2 days. | / | / | We expect a positive correlation of at least 0.3 with SPPB, Gait speed and TUG. We expect a positive correlation of at least 0.2 with MMSE. We expect a negative correlation of at least 0.2 with GDS, FES-I, FFABQ. We expect a negative correlation of at least 0.3 with lying and sitting min and a positive correlation of at least 0.3 with standing, walking, walking episodes, steps. We expect a positive correlation of at least 0.5 with being active outdoors, mean outdoor walking duration, mean outdoor walking distance, outdoor waling episodes and max distance from home. |  | We expect a SRM of at least 0.2 (based on Cohen’s d). |
| LSA | Portuguese | Garcia (2018) | We expect a Cronbach’s alpha of at least 0.70. | We expect an ICC ≥ 0.70 over 7 days. | We expect the SEM to be less than the MIC for LSA (i.e., 2.5-3). | / | We expect a correlation of at least 0.30 b/w LSA and daily steps (accel). |  |  |
| LSA | Portuguese | Simoes (2018) | We expect a Cronbach’s alpha of at least 0.70. | We expect an ICC ≥ 0.70 over 7 days. | We expect the SEM to be less than the MIC for LSA (i.e., 2.5-3). | / | We expect a negative correlation of at least 0.30 between LSA and inactivity, and positive correlation of at least 0.30 between LSA and mod-vig activity. |  |  |
| LSA | Korean | Yang (2017) | / | We expect a kappa value of at least 0.60 over 2 weeks. | / | / | We expect a correlation of at least 0.50 b/w LSA and Functional Ambulation Category (FAC), FIM and mobility subscale of FIM. We expect a positive correlation of at least 0.30 between LSA and EQ-5D, and a negative correlation of 0.30 with GDS. |  |  |
| LSA-C | Swedish | Fristedt (2016) |  | / | / | / | We expect a correlation of at least 0.50 between LSA and SPBB, transportation. We expect a correlation of at least 0.30 between LSA and stair climbing, transfers, food shopping, travel for pleasure, community activities. |  |  |
| LSA-E | Swedish | Fristedt (2016) |  | / | / | / | We expect a correlation of at least 0.50 between LSA and SPBB, transportation. We expect a correlation of at least 0.30 between LSA and stair climbing, transfers, food shopping, travel for pleasure, community activities. |  |  |
| LSA-I | Swedish | Fristedt (2016) |  | / | / | / | We expect a correlation of at least 0.50 between LSA and SPBB, transportation. We expect a correlation of at least 0.30 between LSA and stair climbing, transfers, food shopping, travel for pleasure, community activities. |  |  |
| LSA-M | Swedish | Fristedt (2016) |  | / | / | / | We expect a correlation of at least 0.50 between LSA and SPBB, transportation. We expect a correlation of at least 0.30 between LSA and stair climbing, transfers, food shopping, travel for pleasure, community activities. |  |  |
| LSA | Finnish | Portegijis (2016) |  | / | / | We expect an AUC of at least 0.70 for ADL difficulties at 2-years. |  |  |  |
| LSA | Chinese | Ji (2015) | / | We expect an ICC ≥ 0.70 over 2 weeks. | / | / | We expect a positive correlation of at least 0.5 with the mini-PPT, IADL. We expect a positive correlation of at least 0.3 with ADL, SF-36, MMSE. We expect a negative correlation of at least 0.3 with GDS. |  |  |
| LSA-C | Swedish | Kammerlind (2014) | / | We expect an ICC ≥ 0.70 over 2 weeks. | We expect the ME to be less than the MIC for LSA (i.e., 2.5-3). | / | / | / | / |
| LSA-E | Swedish | Kammerlind (2014) | / | We expect a weighted kappa ≥ 0.70 over 2 weeks. | We expect a % agreement of at least 60% between the 2 time-points. | / | / | / | / |
| LSA-I | Swedish | Kammerlind (2014) | / | We expect a weighted kappa ≥ 0.70 over 2 weeks. | We expect a % agreement of at least 60% between the 2 time-points. | / | / | / | / |
| LSA-M | Swedish | Kammerlind (2014) | / | We expect a weighted kappa ≥ 0.70 over 2 weeks. | We expect a % agreement of at least 60% between the 2 time-points. | / | / | / | / |
| LSA-C | Finnish | Portegijis (2014) | / | We expect an ICC ≥ 0.70 over 2 weeks | / | / | / | / | We expect a correlation of at least 0.20 between LSA and health and 0.3 and mobility criterion. |
| LSA-E | Finnish | Portegijis (2014) | / | We expect an ICC ≥ 0.70 over 2 weeks | / | / | / | / | / |
| LSA-I | Finnish | Portegijis (2014) | / | We expect an ICC ≥ 0.70 over 2 weeks | / | / | / | / | / |
| LSA-M | Finnish | Portegijis (2014) | / | We expect an ICC ≥ 0.70 over 2 weeks | / | / | / | / | / |
| LSA-C | Spanish and Portuguese | Curcio (2013) | / | We expect an ICC ≥ 0.70 over 7-10 days. | / | / | / |  |  |
| LSA-E | Spanish and Portuguese | Curcio (2013) | / | We expect an ICC ≥ 0.70 over 7-10 days. | / | / | / |  |  |
| LSA-I | Spanish and Portuguese | Curcio (2013) | / | We expect an ICC ≥ 0.70 over 7-10 days. | / | / |  |  |  |
| LSA-M | Spanish and Portuguese | Curcio (2013) | / | We expect an ICC ≥ 0.70 over 7-10 days. | / | / |  |  |  |
| LSA | Japanese | Shimada (2010) | / | / | / | We expect a c-index value of at least 0.7 between the LSA and IADL limitations at 1-year. | We expect a positive correlation of at least 0.5 with IADL. | / | / |
| LSA-C | French | Auger (2009) |  | We expect an ICC ≥ 0.70 over the 2-week period. |  |  |  |  |  |
| LSA-E | French | Auger (2009) |  | We expect an ICC ≥ 0.70 over the 2-week period. |  |  |  |  |  |
| LSA-I | French | Auger (2009) |  | We expect an ICC ≥ 0.70 over the 2-week period. |  |  |  |  |  |
| LSA-M | French | Auger (2009) |  | We expect an ICC ≥ 0.70 over the 2-week period. |  |  |  |  |  |
| Modified LSQ | English | Barnes (2007) | / | / | / | / | We expect a positive correlation of at least 0.3 with ADL and with the Rosow-Breslau Scale; 0.5 with IADL. | / | / |
| LSA-C | English | Baker (2003) | / | We expect an ICC ≥ 0.70 over 2 weeks. |  |  | We expect a positive correlation of at least 0.5 with the physical performance measure, SF-12 PCS. We expect a neg corr of at least 0.5 with IADL. We expect a positive correlation of at least 0.3 with SF-12 MC, self-report health. We expect a negative correlation of at least 0.3 with ADL, GDS, comorbidities. |  |  |
| LSA-E | English | Baker (2003) | / | / |  |  | We expect a positive correlation of at least 0.5 with the physical performance measure, SF-12 PCS. We expect a negative correlation of at least 0.5 with IADL. We expect a positive correlation of at least 0.3 with SF-12 MCS, self-report health. We expect a negative correlation of at least 0.3 with ADL, GDS, comorbidities. |  |  |
| LSA-I | English | Baker (2003) | / | We expect an ICC ≥ 0.70 over 6 months. |  |  | We expect a positive correlation of at least 0.5 with the physical performance measure, SF-12 PCS. We expect a neg correlation of at least 0.5 with IADL. We expect a positive correlation of at least 0.3 with SF-12 MCS, self-report health. We expect a negative correlation of at least 0.3 with ADL, GDS, comorbidities. |  |  |
| LSA-M | English | Baker (2003) | / | We expect an ICC ≥ 0.70 over 6 months. |  |  | We expect a positive correlation of at least 0.5 with the physical performance measure, SF-12 PCS. We expect a neg correlation of at least 0.5 with IADL. We expect a positive correlation of at least 0.3 with SF-12 MCS, self-report health. We expect a negative correlation of at least 0.3 with ADL, GDS, comorbidities. |  |  |
| LSQ | English | Stalvey (1999) | / | We expect a weighted kappa ≥ 0.70 over 1 year. | We expect a % agreement of at least 60% for items. | / | We expect a negative correlation of at least 0.1 with visual acuity and a positive correlation of at least 0.1 with contrast sensitivity. We expect a negative correlation of at least 0.2 with useful field of view and ADVS. We expect negative correlations of at. Least 0.3 with comorbidities, depressive symptoms, mental status. We expect a positive correlation of at least 0.1 with driving days/week, at least 0.2 with trips and destinations, and at least 0.3 with miles per week. We expect a positive a correlation of at least 0.5 with the POMA. | / | / |

LSA: Life-Space Assessment; LSA-C: Life-Space Assessment-Composite; LSA-E: Life-Space Assessment-Equipment; LSA-M: Life-Space Assessment-Maximal; LSA-I: Life-Space Assessment-Independent; LSQ: Life-Space Questionnaire; ADL: Activities of Daily Living; CESD-10: Center for Epidemiologic Studies Depression Scale- 10 items; CI: Confidence Interval; EQ-5D: EuroQol 5-Dimensions; FAC: Functional Ambulation Category; FES-I: Falls Efficacy Scale International; FFABQ: Fear of Falling Avoidance Behaviour Questionnaire; FIM: Functional Independence Measure; GDS: Geriatric Depression Scale; ICC: Intraclass Correlation Coefficient; IADL: Instrumental Activities of Daily Living; MAT-sf: Mobility Assessment Tool short form; MFAQ: Multidimensional Functional Assessment Questionnaire; Mini PPT: Mini Physical Performance Test; MMSE: Mini Mental State Examination; OR: Odds Ratio; PADL: Physical Activities of Daily Living; POMA: Performance Oriented Mobility Assessment; ROB: Risk of Bias; SE: Standard Error; SEM: Standard Error of Measurement; SF-36: Short Form Health Survey 36 items; SF-12 MCS: Short Form Health Survey 12 items Mental Component Scale; SF-12 PCS: Short Form Health Survey 12 items Physical Component Scale; SPPB: Short Physical Performance Battery; SRM: Standardized Response Mean; TUG: Timed-Up and Go
