# Supplementary material for: Assessing the measurement properties of life-space mobility measures in community-dwelling older adults: a systematic review
Source: Age Ageing. 2023 Oct 30;52(Suppl 4):iv86–99. doi: 10.1093/ageing/afad119 (PMC10615067; doi:10.1093/ageing/afad119)
Supplement: aa-23-0362-File004_afad119 [file aa-23-0362-file004_afad119.docx]

**Appendix C. Characteristics of Life-Space Mobility Measures Included in the Review**

| **Measure** | **Developer** | **Number of items** | **Response options** | **Total score** | **Total Score interpretation** | **Subscale Scores** | **Recall period** |
| --- | --- | --- | --- | --- | --- | --- | --- |
| Life Space Assessment (LSA) | Baker (2003) | 15 (5 life-space levels + 5 frequency + 5 independence) | Life-space levels: Yes/No.  Frequency: less than 1/week, 1-3 times/week, 4-6 times/ week, daily.  Independence: Personal assistance, Equipment only, No equipment or personal assistance. | Composite Score (LSA-C)  0-120 (obtained from multiplying level, frequency and independence and summing them up) | Life-space level attained considering degree of independence and frequency | Life Space Assessment with Equipment (LSA-E) score 0 to 5  Independent Life Space Assessment (LSA-I) score 0 to 5  Maximum Life Space Assessment (LSA-M) score 0 to 5 | Past 4-weeks |
| Modified Life Space Assessment (Modified LSA) | Ullrich (2019) | 15 (5 life-space levels + 5 frequency + 5 independence) | Life-space levels: Yes/No.  Frequency:1-3 times/week, 4-6 times/ week, daily.  Independence: Personal assistance, Equipment only, No equipment or personal assistance. | Composite Score  0-90 (obtained from multiplying level, frequency and independence and summing them up) | Life-space level attained considering degree of independence and frequency | Life Space Assessment with Equipment  score 0 to 5  Independent Life Space Assessment score 0 to 5  Maximum Life Space Assessment score 0 to 5 | Past 1-week |
| Life Space Questionnaire (LSQ) | Stalvey (1999) | 9 | Yes/No | 0-9 (obtained by taking the sum of responses) | Larger score = larger life-space | / | Past 3 days |
| Modified Life Space Questionnaire (Modified LSQ) | Barnes (2007) | 6 | Yes/No | 0-6 (obtained by taking the sum of responses) | Larger score = larger life-space | / | Past 1-week |
