# Supplementary material for: Assessing the measurement properties of life-space mobility measures in community-dwelling older adults: a systematic review
Source: Age Ageing. 2023 Oct 30;52(Suppl 4):iv86–99. doi: 10.1093/ageing/afad119 (PMC10615067; doi:10.1093/ageing/afad119)
Supplement: aa-23-0362-File005_afad119 [file aa-23-0362-file005_afad119.docx]

**Appendix D. Life-Space Measurement Review-Summary of Finding Tables for the modified Life-Space Assessment (LSA), LSA Subscales, the Life-Space Questionnaire (LSQ), and the modified LSQ.**

Table. Summary of findings for reliability

| **Reliability** | **# of tests/hypotheses** | **Summary or pooled result** | **Overall rating** | **Quality of evidence** |
| --- | --- | --- | --- | --- |
| Modified LSA-C | 2 | 100% of hypotheses were confirmed. ICC ranges from 0.89-0.91 | Sufficient | Moderate |
| LSA-E | 4 | 75% of hypotheses were confirmed; ICC ranges from 0.37-0.76 | Sufficient; | Low |
| Modified LSA-E | 2 | 50% of hypotheses were confirmed. ICC ranges from 0.65 to 0.78 | Sufficient; inconsistent | Very low |
| LSA-I | 5 | 80% of hypotheses were confirmed; ICC ranges from 0.63-0.94 | Sufficient | Low |
| Modified LSA-I | 2 | 100% of hypotheses were confirmed. ICC ranges from 0.81-0.91 | Sufficient | Low |
| LSA-M | 5 | 20% of hypotheses were confirmed; ICC ranges from 0.49-0.81 | Insufficient; inconsistent | Very low |
| Modified LSA-M | 2 | 50% of hypotheses were confirmed; ICC ranges from 0.64-0.80 | Sufficient; inconsistent | Very low |
| LSQ* | 1 | 100% of hypotheses were confirmed; Weighted kappa=0.80 | Sufficient | Very low |

*only 1 study

ICC: Intraclass Correlation Coefficient; LSA: Life-Space Assessment; LSA-C: Life-Space Assessment-Composite; LSA-E: Life-Space Assessment-Equipment; LSA-M: Life-Space Assessment-Maximal; LSA-I: Life-Space Assessment-Independent; LSQ: Life-Space Questionnaire.

Table. Summary of findings for measurement error

| **Measurement Error** | **# of tests/hypotheses** | **Summary or pooled result** | **Overall rating** | **Quality of evidence** |
| --- | --- | --- | --- | --- |
| LSA-E | 2 | 50% of hypotheses were confirmed; 59-80% agreement | Sufficient; inconsistent | Low |
| LSA-I | 2 | 100% of hypotheses were confirmed; 62-80% agreement | Sufficient | Moderate |
| LSA-M | 2 | 100% of hypotheses were confirmed; 62-81% agreement | Sufficient | Moderate |
| LSQ* | 1 | 100% of hypotheses were confirmed; 70-90% agreement | Sufficient | Very low |

*only 1 study

LSA: Life-Space Assessment; LSA-C: LSA-E: Life-Space Assessment-Equipment; LSA-M: Life-Space Assessment-Maximal; LSA-I: Life-Space Assessment-Independent; LSQ: Life-Space Questionnaire.

Table. Summary of findings for content validity

| **Content Validity** | **Overall rating** | **Quality of evidence** |
| --- | --- | --- |
| Modified LSA | Sufficient | Moderate* |
| LSQ | Inconsistent | Very low* |
| Modified LSQ | Inconsistent | Very low* |

* Only considers risk of bias, inconsistency, and indirectness in GRADE.

Note: Content validity results for the LSA are included in Table 3.

LSA: Life-Space Assessment; LSQ: Life-Space Questionnaire.

Table. Summary of findings for convergent validity

| **Convergent validity** | **# of tests/hypotheses** | **Summary or pooled result** | **Overall rating** | **Quality of evidence** |
| --- | --- | --- | --- | --- |
| Modified LSA-C | 25 | 68% of hypotheses were confirmed | Sufficient; inconsistent | Moderate |
| LSA-E | 15 | 40% of hypotheses were confirmed | Insufficient; inconsistent | Moderate |
| Modified LSA-E | 25 | 76% of hypotheses were confirmed | Sufficient | High |
| LSA-I | 15 | 73% of hypotheses were confirmed | Sufficient; inconsistent | Moderate |
| Modified LSA-I | 25 | 40% of hypotheses confirmed | Insufficient; inconsistent | Low |
| LSA-M | 15 | 0% of hypotheses were confirmed | Insufficient | High |
| Modified LSA-M | 25 | 4% of hypotheses were confirmed | Insufficient | High |
| LSQ | 12 | 25% of hypotheses were confirmed | Insufficient | Moderate |
| Modified LSQ | 3 | 0% of hypotheses were confirmed | Insufficient | High |

LSA: Life-Space Assessment; LSA-C: LSA-E: Life-Space Assessment-Equipment; LSA-M: Life-Space Assessment-Maximal; LSA-I: Life-Space Assessment-Independent; LSQ: Life-Space Questionnaire.

Table. Summary of findings for responsiveness

| **Responsiveness** | **Number of tests** | **Summary or pooled result** | **Overall rating** | **Quality of evidence** |
| --- | --- | --- | --- | --- |
| Modified LSA-C | 2 | 100% of hypotheses were confirmed SRM: 0.70- 0.80 | Sufficient | Low |
| Modified LSA-E | 2 | 100% of hypotheses were confirmed; SRM: 0.33- 0.35 | Sufficient | Low |
| Modified LSA-I | 2 | 100% of hypotheses were confirmed; SRM: 0.43-0.46 | Sufficient | Low |
| Modified LSA-M | 2 | 100% of hypotheses were confirmed; SRM: 0.48-0.60 | Sufficient | Low |

SRM: Standardized Response Mean; LSA: Life-Space Assessment; LSA-C: LSA-E: Life-Space Assessment-Equipment; LSA-M: Life-Space Assessment-Maximal; LSA-I: Life-Space Assessment-Independent; LSQ: Life-Space Questionnaire.
