# Supplementary material for: Assessing the measurement properties of life-space mobility measures in community-dwelling older adults: a systematic review
Source: Age Ageing. 2023 Oct 30;52(Suppl 4):iv86–99. doi: 10.1093/ageing/afad119 (PMC10615067; doi:10.1093/ageing/afad119)
Supplement: aa-23-0362-File006_afad119 [file aa-23-0362-file006_afad119.docx]

**Appendix E. Life-Space Measurement Review-GRADE**

Table. Overview of GRADE for each property for the LSA composite score

| **Property** | **Pooled Results** | **Total Sample Size** | **Risk of Bias** | **Inconsistency** | **Imprecision** | **Indirectness** | **GRADE** |
| --- | --- | --- | --- | --- | --- | --- | --- |
| Internal consistency | 2+ Sufficient (Cronbach alpha: 0.8-0.92) | 142 | No | No | No | No | High |
| Reliability | 10+ Sufficient *see forest plot | 978 | No | No | No | No | High |
| Measurement error | 3- Insufficient (SEM ranges from 4.12-9.1) | 440 | No | No | No | No | High |
| Predictive validity | 2+ Sufficient | 1191 | No | No | No | No | High |
| Convergent validity | 28+; 8- Sufficient | 4944 | No | No | No | No | High |
| Known groups validity | 2+;1? (sufficient; inconsistent) | 276 | Very serious | Serious | No | Very serious | Very low |
| Responsiveness | 2+;2- (sufficient; inconsistent) | 1036 | No | Serious | No | No | Moderate |

Table. Overview of GRADE for each property for the modified LSA composite score

| **Property** | **Pooled Results** | **Total Sample Size** | **Risk of Bias** | **Inconsistency** | **Imprecision** | **Indirectness** | **GRADE** |
| --- | --- | --- | --- | --- | --- | --- | --- |
| Reliability | 2+ Sufficient | 157 | Serious | No | No | No | Moderate |
| Convergent validity | 17+; 8- Sufficient; inconsistent | 182 | No | Serious | No | No | Moderate |
| Responsiveness | 2+ Sufficient | 85 | Serious | No | Serious | No | Low |

Table. Overview of GRADE for each property for the LSA with equipment subscale (LSA-E) score

| **Property** | **Pooled Results** | **Total Sample Size** | **Risk of Bias** | **Inconsistency** | **Imprecision** | **Indirectness** | **GRADE** |
| --- | --- | --- | --- | --- | --- | --- | --- |
| Reliability | 3+; 1- Sufficient; ICC ranges from 0.37-0.76 | 416 | Very serious | No | No | No | Low |
| Measurement error | 1+; 1-Sufficient; inconsistent. 59-80% agreement | 337 | Serious | Serious | No | No | Low |
| Convergent validity | 6+; 9- Insufficient; inconsistent. | 618 | No | Serious | No | No | Moderate |

Table. Overview of GRADE for each property for the modified LSA-E subscale score

| **Property** | **Pooled Results** | **Total Sample Size** | **Risk of Bias** | **Inconsistency** | **Imprecision** | **Indirectness** | **GRADE** |
| --- | --- | --- | --- | --- | --- | --- | --- |
| Reliability | 1+; 1- Sufficient; inconsistent. ICC ranges from 0.65 to 0.78 | 157 | Very serious | Serious | No | No | Very low |
| Convergent validity | 19+; 6- Sufficient | 182 | No | No | No | No | High |
| Responsiveness | 2+ Sufficient. SRM 0.33- 0.35 | 85 | Serious | No | Serious | No | Low |

Table. Overview of GRADE for each property for the Independent LSA (LSA-I) subscale score

| **Property** | **Pooled Results** | **Total Sample Size** | **Risk of Bias** | **Inconsistency** | **Imprecision** | **Indirectness** | **GRADE** |
| --- | --- | --- | --- | --- | --- | --- | --- |
| Reliability | 4+;1- Sufficient. ICC ranges from 0.63-0.94 | 722 | Very serious | No | No | No | Low |
| Measurement error | 2+ Sufficient 62-80% agreement | 337 | Serious | No | No | No | Moderate |
| Convergent validity | 11+; 4- Sufficient; inconsistent. | 618 | No | Serious | No | No | Moderate |

Table. Overview of GRADE for each property for the modified LSA-I subscale score

| **Property** | **Pooled Results** | **Total Sample Size** | **Risk of Bias** | **Inconsistency** | **Imprecision** | **Indirectness** | **GRADE** |
| --- | --- | --- | --- | --- | --- | --- | --- |
| Reliability | 2+ Sufficient. ICC ranges from 0.81-0.91 | 157 | Very serious | No | No | No | Low |
| Convergent validity | 10+; 15- Insufficient; inconsistent. Correlations with performance-based measures: 0.52 (TUG and SPPB) – 0.56 (Gait speed) | 182 | No | Very serious | No | No | Low |
| Responsiveness | 2+ Sufficient 0.43-0.46 | 85 | Serious | No | Serious | No | Low |

Table. Overview of GRADE for each property for the Maximum LSA (LSA-M) subscale score

| **Property** | **Pooled Results** | **Total Sample Size** | **Risk of Bias** | **Inconsistency** | **Imprecision** | **Indirectness** | **GRADE** |
| --- | --- | --- | --- | --- | --- | --- | --- |
| Reliability | 1+; 4- Insufficient; inconsistent. ICC ranges form 0.49-0.81 | 722 | Very serious | Very serious | No | No | Very low |
| Measurement error | 2+ Sufficient 62-81% agreement | 337 | Serious | No | No | No | Moderate |
| Convergent validity | 15- Insufficient. | 618 | No | No | No | No | High |

Table. Overview of GRADE for each property for the modified LSA-M subscale score

| **Property** | **Pooled Results** | **Total Sample Size** | **Risk of Bias** | **Inconsistency** | **Imprecision** | **Indirectness** | **GRADE** |
| --- | --- | --- | --- | --- | --- | --- | --- |
| Reliability | 1+; 1- Sufficient; inconsistent. ICC ranges from 0.64-0.80 | 157 | Very serious | Serious | No | No | Very low |
| Convergent validity | 24-; 1+ Insufficient. Correlations with performance-based measures: 0.05 (SPPB) – 0.28 (SPPB) | 182 | No | No | No | No | High |
| Responsiveness | 2+ Sufficient SRM ranges from 0.48-0.6 | 85 | Serious | No | Serious | No | Low |

Table. Overview of GRADE for each property for the LSQ score

| **Property** | **Pooled Results** | **Total Sample Size** | **Risk of Bias** | **Inconsistency** | **Imprecision** | **Indirectness** | **GRADE** |
| --- | --- | --- | --- | --- | --- | --- | --- |
| Reliability | Weighted kappa=0.80 (+) Sufficient | 200 | Extremely serious | No | No | Serious | Very low |
| Measurement error | Agreement > 70% (+) Sufficient | 200 | Extremely serious | No | No | No | Very low |
| Convergent validity | (9-, 3+); Insufficient | 242 | Serious | No | No | No | Moderate |

Table. Overview of GRADE for each property for the modified LSQ

| **Property** | **Pooled Results** | **Total Sample Size** | **Risk of Bias** | **Inconsistency** | **Imprecision** | **Indirectness** | **GRADE** |
| --- | --- | --- | --- | --- | --- | --- | --- |
| Convergent validity | 3- Insufficient | 909 | No | No | No | No | High |
