# Supplementary material for: Assessing the measurement properties of life-space mobility measures in community-dwelling older adults: a systematic review
Source: Age Ageing. 2023 Oct 30;52(Suppl 4):iv86–99. doi: 10.1093/ageing/afad119 (PMC10615067; doi:10.1093/ageing/afad119)
Supplement: aa-23-0362-File007_afad119 [file aa-23-0362-file007_afad119.docx]

World Health Organization: *Measurement of Healthy Ageing.*

**Assessing the Measurement Properties of Life-Space Mobility Measures in Community-Dwelling Older Adults: A Systematic Review**

SUPPLEMENTARY DATA

**Appendix F. Life-Space Measurement Review-Results Tables**

Table. Psychometric Properties of the LSA-composite score (n=14)

| **Measure** | **Language** | **Author (year)** | **Sample size** | **ROB** | **Internal consistency** | **ROB rating** | **Reliability** | **ROB** | **Measurement error** | **ROB** | **Predictive validity** | **ROB** | **Convergent validity** | **ROB** | **Known-groups validity** | **ROB** | **Responsiveness** |
| --- | --- | --- | --- | --- | --- | --- | --- | --- | --- | --- | --- | --- | --- | --- | --- | --- | --- |
| LSA | Chinese | Tseng (2020) | 225  (40 for reliability) |  |  | Adequate | 2 weeks: ICC= 0.88 (95% CI: 0.78-0.94) (+) |  |  |  |  | Very good | With PADL : r=0.53 (p<0.01); IADL: r=0.69 (p<0.01); MFAQ: r=0.66 (p<0.01); CESD10: r=-0.54 (p<0.01); SF36 general health: r=0.68 (p <0.01) (5+) |  |  |  |  |
| LSA | English | McCrone (2019) | 276 for known groups; 273 for convergent validity; 228 for responsiveness |  |  |  |  |  |  |  |  | Very good | POMA: Kendall tau-b= 0.37 (p<0.001) (-) | doubtful | *P <0.001. Mean difference between OP and DNH cohorts: 14.1 (95% CI = 9.2 to 19.0)* points; B/w OP and DH cohorts= 17.3 (95% CI = 12.6 to 22.1)* points; B/w the DNH and DH cohorts =3.3 [95% CI = −0.4 to 6.9] points (2+, 1?) | Very good | LSA change score and POMA1 change score over treatment (median time of 53 days): Kendall tau-b=0.27 (p<0.001). SRM = 0.60; P < .001 (1+, 1-) |
| LSA | Portuguese | Garcia (2018) | 62 | Very good | Cronbach’s α = 0.80 (+) | Very good | 7 days: ICC (95% CI) = 0.95(0.91-0.97)  (+) | Very good | SEM (%) =3.7 (3%)  (-) |  |  | Adequate | With daily step (accel.): r = 0.43, P= 0.01 (+) |  |  |  |  |
| LSA | Portuguese | Simoes (2018) | 80 | Very good | Cronbach’s α = 0.92 (0.91–0.92) (+) | Adequate | 7 days: ICC (95%CI) =0.97 (0.95-0.98) (+) | Adequate | SEM = 4.12 (-) |  |  | Adequate | With inactivity (accel): r= -0.63 (p<0.001); With moderate-to-vigorous (accel): r= 0.49 (p<0.001) (2+) |  |  |  |  |
| LSA | Korean | Yang (2017) | 34 |  |  | Inadequate | 2 weeks: Kappa=0.993 (p<0.01) (+) |  |  |  |  | Doubtful | With FAC: rho=0.848, (p<0.01); FIM: rho=0.765 (p<0.01); mobility subscale of the FIM: rho=0.764 (p<0.01); EQ-5D: rho=0.506 (p<0.01); GDS: rho=-0.657 (p<0.01) (5+) |  |  |  |  |
| LSA-C | Swedish | Fristedt (2016) | 312 |  |  |  |  |  |  |  |  | Very good | SPPB total score: rho= 0.57; transportation rho =0.63; stair climbing: rho=0.45; transfers: rho=0.28 ; food shopping: rho =0.55; travel for pleasure: rho =0.42; community activities: rho=0.38 (6+, 1-) |  |  |  |  |
| LSA | Finnish | Portegijis (2016) | Baseline: 848; longitudinal: 755 |  |  |  |  |  |  | Very good | Baseline LSA scores identifying those who develop ADL difficulties at 2-year follow-up: AUC= 0.79 (+) |  |  |  |  |  |  |
| LSA | Chinese | Ji (2015) | 100 (40 for reliability) |  |  | Doubtful | 2-weeks: ICC=0.76 (+) |  |  |  |  | Very good | *= P<0.001. ADL: r=0.074; IADL:r=0.433*; Mini-PPT: r=0.567*;SF36: r=0.704*;GDS:r= -0.544*; MMSE: r=0.424* (4+,2-) |  |  |  |  |
| LSA-C | Swedish | Kammerlind (2014) | 298 |  |  | Doubtful | 2 weeks:  ICC=0.84 (0.81 to 0.87) (+) | Doubtful | SEM=9.1 (-) |  |  |  |  |  |  |  |  |
| LSA-C | Finnish | Portegijis (2014) | 1-year: 808; Reliability: 39 |  |  | Adequate | 2 weeks: ICC (95% CI)= 0.72 (0.52-0.84) (+) |  |  |  |  |  |  |  |  | Very good | 1-year follow-up b/w Life space mobility score and health criterion: rho=-0.223 (p<0.001); and criterion mobility: rho=-0.217 (p<0.001) (1+;1-) |
| LSA-C | Spanish and Portuguese | Curcio (2013) | 300 (39 for reliability) |  |  | Adequate | 7-10 days: ICC (95 % CI) = 0.70 (0.49–0.83) (+) |  |  |  |  |  |  |  |  |  |  |
| LSA | Japanese | Shimada (2010) | Baseline: 2404; longitudinal: 436 |  |  |  |  |  |  | Inadequate | C-index = 0.84 b/w LSA and IADL limitations at 1 year(+) | Very good | IADLs: r=0.57, P<0.001 (+) |  |  |  |  |
| LSA-C | French | Auger (2009) | 40 |  |  | Adequate | 7-10 days: ICC=0.87 (0.69-0.92) (+) |  |  |  |  |  |  |  |  |  |  |
| LSA-C | English | Baker (2003) | 306 |  |  | Adequate | 2 weeks: ICC= 0.96 (0.95–0.97)  (+) |  |  |  |  | Adequate | Physical performance measure: rho=0.603; ADL: rho=-0.396; IADLS: rho= –0.392; SF12PCS: rho=0.437; SF12 MCS: rho= 0.257; GDS: rho=–0.411; comorbid cond.: rho=–0.194; self-reported health: rho=0.421  (4+,4-) |  |  |  |  |
| **Total** | | | **2+ Sufficient (Cronbach alpha: 0.8-0.92)** | | | **10+ Sufficient *see forest plot (10+ include kappa)** | | **3- Insufficient (SEM ranges from 4.12-9.1)** | | **2+ Sufficient (Able to identify those with declines in ADLs/IADLs)** | | **28+; 8- Sufficient *see forest plots** Correlations with performance-based measures: 0.37 (POMA) -0.60 (SPPB)  Correlations with health-related quality of life: 0.26 (SF-12 mental component) – 0.70 (SF-36) | | **2+;1? (sufficient; inconsistent)** | | **2+;2- (sufficient; inconsistent)** | |

ADL: Activities of Daily Living; CESD-10: Center for Epidemiologic Studies Depression Scale- 10 items; CI: Confidence Interval; EQ-5D: EuroQol 5-Dimensions; FAC: Functional Ambulation Category; FES-I: Falls Efficacy Scale International; FFABQ: Fear of Falling Avoidance Behaviour Questionnaire; FIM: Functional Independence Measure; GDS: Geriatric Depression Scale; ICC: Intraclass Correlation Coefficient; IADL: Instrumental Activities of Daily Living; LSA: Life-Space Assessment; LSA-C: Life-Space Assessment-Composite; MAT-sf: Mobility Assessment Tool short form; MFAQ: Multidimensional Functional Assessment Questionnaire; Mini PPT: Mini Physical Performance Test; MMSE: Mini Mental State Examination; OR: Odds Ratio; PADL: Physical Activities of Daily Living; POMA: Performance Oriented Mobility Assessment; ROB: Risk of Bias; SE: Standard Error; SEM: Standard Error of Measurement; SF-36: Short Form Health Survey 36 items; SF-12 MCS: : Short Form Health Survey 12 items Mental Component Scale; SF-12 PCS: Short Form Health Survey 12 items Mental Component Scale; SPPB: Short Physical Performance Battery; SRM: Standardized Response Mean; TUG: Timed-Up and Go

Table. Psychometric Properties of the modified LSA composite score (LSA-C) (n=2)

| **Measure** | **Language** | **Author (year)** | **Sample size** | **ROB rating** | **Reliability** | **ROB** | **Convergent validity** | **ROB** | **Responsiveness** |
| --- | --- | --- | --- | --- | --- | --- | --- | --- | --- |
| Modified LSA-C | German | Ullrich (2021) | 65 for convergent validity; 55 for reliability; 32 for responsiveness | Doubtful | Within 3 days: ICC (95% CI)= 0.89 (0.819-0.934) (+) | Adequate | With SPPB: rho=0.55**; FFABQ: rho=-0.38**; Walking episodes: rho=0.38**;Steps: rho-0.60**; mean outdoor walking duration: rho=0.49**; mean outdoor walking distance: rho=0.49**; outdoor walking episodes: rho=0.49**. (*p<0.05; **p<0.01) (4+;3-) | Doubtful | Post-intervention 12 -weeks: SRM= 0.70 (+) |
| Modified LSA-C | German | Ullrich (2019) | 117 for convergent validity; 102 for reliability; 53 for responsiveness | Doubtful | Within 2 days: ICC (95% CI)=0.91 (0.87-0.94) (+) | Very good | With SPPB: rho= 0.39**; Gait speed: rho=0.41**; TUG: rho=-0.40**; MMSE: rho=0.18; GDS: rho=-0.11; FES-I: rho=-0.24**; FFABQ: rho=-0.38**; Lying min: rho=-0.13; sitting min: rho=-0.12; standing min: rho=0.41**; walking min: rho=0.55**;walking episodes: rho=0.40**; steps: rho=0.59**; being active outdoors: rho=0.53**; mean outdoor walking duration: rho=0.54**; mean outdoor walking distance: rho=0.54**; outdoor walking episodes: rho=0.54**; max distance from home: rho=0.52**(**=p<0.01) (13+, 5- ) | Doubtful | LSACIC: b/w baseline and post-intervention 12- weeks p<0.001 SRM=0.80 (+) |
| Total | | |  | 2+ Sufficient ICC ranges from 0.89-0.91 | | 17+,8- Sufficient; inconsistent | | 2+ Sufficient SRM=0.70-0.80 | |

Table. Psychometric Properties of the LSA with equipment (LSA-E) score (n=6)

| **Measure** | **Language** | **Author (year)** | **Sample size** | **ROB** | **Reliability** | **ROB** | **Measurement error** | **ROB** | **Convergent validity** |
| --- | --- | --- | --- | --- | --- | --- | --- | --- | --- |
| LSA-E | Swedish | Fristedt (2016) | 312 |  |  |  |  | Very good | SPBB total score: rho= 0.47; transportation rho =0.64; stair climbing: rho=0.28; transfers: rho=0.28 ; food shopping: rho =0.55; travel for pleasure: rho =0.41; community activities: rho=0.35 (5+, 2-) |
| LSA-E | Swedish | Kammerlind (2014) | 298 | Doubtful | 2 weeks: Weighted kappa=0.86 (0.81–0.91) (+) | Doubtful | % 80 agreement (+) |  |  |
| LSA-E | Finnish | Portegijis (2014) | 1-year: 808; Reliability: 39 | Inadequate | 2 weeks: ICC (95 % CI)=0.72(0.53-0.84) (+) | Adequate | 59% agreement (-) |  |  |
| LSA-E | Spanish and Portuguese | Curcio (2013) | 300 (39 for reliability) | Inadequate | 7-10 days: ICC (95 % CI ) =0.37(0.07–0.61) (-) |  |  |  |  |
| LSA-E | French | Auger (2009) | 40 | Inadequate | 7-10 days: ICC=0.76 (0.54-0.87) (+) |  |  |  |  |
| LSA-E | English | Baker (2003) | 306 |  |  |  |  | Adequate | Physical performance measure: rho=0.463; ADL: rho=–0.246; IADLS: rho=–0.268; SF12PCS: rho=0.310; SF12 MCS: rho=0.187; GDS: rho=–0.300; Comorbid cond: rho=–0.149; self-reported health: rho=0.273.  (1+,7-) |
| **Total** | | | **3+; 1- Sufficient. ICC ranges from 0.37-0.76** | | | **1+; 1-Sufficient; inconsistent. 59-80% agreement** | | **6+; 9- Insufficient; inconsistent.** | |

ADL: Activities of Daily Living; CI: Confidence Interval; FES-I: Falls Efficacy Scale International; FFABQ: Fear of Falling Avoidance Behaviour Questionnaire; GDS: Geriatric Depression Scale; ICC: Intraclass Correlation Coefficient; IADL: Instrumental Activities of Daily Living; LSA-CI: Life-Space Assessment-Cognitive Impairment; LSA-E: Life-Space Assessment-Assistive; MMSE: Mini Mental State Examination; ROB: Risk of Bias; SF-12 MCS: Short Form Health Survey 12 items Mental Component Scale; SF-12 PCS: Short Form Health Survey 12 items Mental Component Scale; SPPB: Short Physical Performance Battery; SRM: Standardized Response Mean; TUG: Timed-Up and Go

Table. Psychometric Properties of the modified LSA equipment score (LSA-E) (n=2)

| **Measure** | **Language** | **Author (year)** | **Sample size** | **ROB** | **Reliability** | **ROB** | **Convergent validity** | **ROB** | **Responsiveness** |
| --- | --- | --- | --- | --- | --- | --- | --- | --- | --- |
| Modified LSA-E | German | Ullrich (2021) | 65 for convergent validity; 55 for reliability; 32 for responsiveness | Inadequate | Within 3 days: ICC (95% CI)= 0.777 (0.645-0.863) (+) | Adequate | With SPPB: rho=0.41**; FFABQ: rho=-0.28*; Walking episodes: rho=0.39**;Steps: rho-0.56**; mean outdoor walking duration: rho=0.53**; mean outdoor walking distance: rho=0.53**; outdoor walking episodes: rho=0.54**. (*p<0.05; **p<0.01) (5+,2-) | Doubtful | Post-intervention; 12 weeks: SRM= 0.33 (+) |
| Modified LSA-E | German | Ullrich (2019) | 117 for convergent validity; 102 for reliability; 53 for responsiveness | Inadequate | Within 2 days: ICC=0.65 (0.53-0.75) (-) | Very good | With SPPB: rho= 0.30*; Gait speed: rho=0.27**; TUG: rho=-0.38**; MMSE: rho=0.21*; GDS: rho=-0.11; FES-I: rho=-0.12; FFABQ: rho=-0.35**; Lying min: rho=-0.25**; sitting min: rho=-0.04; standing min: rho=0.53**; walking min: rho=0.58**;walking episodes: rho=0.42**; steps: rho=0.60**; being active outdoors: rho=0.63**; mean outdoor walking duration: rho=0.62**; mean outdoor walking distance: rho=0.63**; outdoor walking episodes: rho=0.62**; max distance from home: rho=0.63**(*=p<0.05;**=p<0.01) (14+,4-) | Doubtful | Baseline vs post-intervention; 12 weeks: p<0.001 SRM=0.35 (+) |
| **Total** | | | **1+; 1- Sufficient; inconsistent. ICC=0.65-0.78** | | | **19+; 6- Sufficient** | | **2+ Sufficient.** | |

Table. Psychometric Properties of the LSA-independent score (LSA-I) (n=6)

| **Measure** | **Language** | **Author (year)** | **Sample size** | **ROB** | **Reliability** | **ROB** | **Measurement error** | **ROB** | **Convergent validity** |
| --- | --- | --- | --- | --- | --- | --- | --- | --- | --- |
| LSA-I | Swedish | Fristedt (2016) | 312 |  |  |  |  | Very good | SPBB total score: rho= 0.63; transportation rho =0.66; stair climbing: rho=0.42; transfers: rho=0.30 ; food shopping: rho =0.58; travel for pleasure: rho =0.44; community activities: rho=0.35 (7+) |
| LSA-I | Swedish | Kammerlind (2014) | 298 | Doubtful | 2 weeks: Weighted kappa = 0.94 (0.92–0.96) (+) | Doubtful | % 80 agreement (+) |  |  |
| LSA-I | Finnish | Portegijis (2014) | 1-year: 808; Reliability: 39 | Inadequate | 2 weeks: ICC (95% CI) =0.86 (0.75-0.92) (+) | Adequate | 62 % agreement (+) |  |  |
| LSA-I | Spanish and Portuguese | Curcio (2013) | 300 (39 for reliability) | Inadequate | 7-10 days: ICC (95 %CI) = 0.63 (0.38–0.77) (-) |  |  |  |  |
| LSA-I | French | Auger (2009) | 40 | Inadequate | 7-10 days: ICC (95% CI) =0.84 (0.69-0.92) (+) |  |  |  |  |
| LSA-I | English | Baker (2003) | 306 | Inadequate | 2 weeks: ICC (95% CI) = 0.81 (0.77–0.84) (+) |  |  | Adequate | Physical performance measure: rho=0.592; ADL: rho=–0.394; IADLS: rho=–0.391; SF12PCS: rho=0.453; SF12 MCS: rho=0.207; GDS: rho=–0.363; Comorbid cond: rho=–0.194; self-reported health: rho=0.358. (4+, 4-) |
| **Total** | | | **4+;1- Sufficient. ICC ranges from 0.63-0.94** | | | **2+ Sufficient 62-80% agreement** | | **11+; 4- Sufficient; inconsistent.** | |

ADL: Activities of Daily Living; CI: Confidence Interval; FES-I: Falls Efficacy Scale International; FFABQ: Fear of Falling Avoidance Behaviour Questionnaire; GDS: Geriatric Depression Scale; ICC: Intraclass Correlation Coefficient; IADL: Instrumental Activities of Daily Living; LSA-I: Life-Space Assessment-Independent; MAT-sf: Mobility Assessment Tool short form; MMSE: Mini Mental State Examination; OR: Odds Ratio; ROB: Risk of Bias; SF-12 MCS: Short Form Health Survey 12 items Mental Component Scale; SF-12 PCS: Short Form Health Survey 12 items Mental Component Scale; SPPB: Short Physical Performance Battery; SRM: Standardized Response Mean; TUG: Timed-Up and Go

Table. Psychometric Properties of the modified LSA independent score (LSA-I) (n=2)

| **Measure** | **Language** | **Author (year)** | **Sample size** | **ROB** | **Reliability** | **ROB** | **Convergent validity** | **ROB** | **Responsiveness** |
| --- | --- | --- | --- | --- | --- | --- | --- | --- | --- |
| Modified LSA-I | German | Ullrich (2021) | 65 for convergent validity; 55 for reliability; 32 for responsiveness | Inadequate | Within 3 days: ICC (95% CI)= 0.814 (0.7-0.887) (+) | Adequate | With SPPB: rho=0.55**; FFABQ: rho=-0.54**; Walking episodes: rho=0.29*;Steps: rho-0.41**; mean outdoor walking duration: rho=0.32*; mean outdoor walking distance: rho=0.23; outdoor walking episodes: rho=0.21. (*p<0.05; **p<0.01) (3+,4-) | Doubtful | Post-intervention; 12 weeks: SRM= 0.46 (+) |
| Modified LSA-I | German | Ullrich (2019) | 117 for convergent validity; 102 for reliability; 53 for responsiveness | Inadequate | Within2 days: ICC (95% CI)= 0.91 (0.86-0.94) (+) | Very good | With SPPB: rho= 0.52**; Gait speed: rho=0.56**; TUG: rho=-0.52**; MMSE: rho=0.02; GDS: rho=-0.02; FES-I: rho=-0.25**; FFABQ: rho=-0.44**; Lying min: rho=-0.14; sitting min: rho=-0.07; standing min: rho=0.23*; walking min: rho=0.51**;walking episodes: rho=0.42**; steps: rho=0.53**; being active outdoors: rho=0.33**; mean outdoor walking duration: rho=0.37**; mean outdoor walking distance: rho=0.33**; outdoor walking episodes: rho=0.32**; max distance from home: rho=0.32**(*=p<0.05;**=p<0.01) (7+,11-) | Doubtful | Baseline and post-intervention; 12 weeks: p=0.001 SRM=0.43 (+) |
| **Total** | | | **2+ Sufficient ICC+0.81-0.91** | | | **10+; 15- Insufficient; inconsistent.** Correlations with performance-based measures: 0.52 (TUG and SPPB) – 0.56 (Gait speed) | | **2+ Sufficient 0.43-0.46** | |

Table. Psychometric Properties of the LSA-maximal score (LSA-M) (n=6)

| **Measure** | **Language** | **Author (year)** | **Sample size** | **ROB** | **Reliability** | **ROB** | **Measurement error** | **ROB** | **Convergent validity** |
| --- | --- | --- | --- | --- | --- | --- | --- | --- | --- |
| LSA-M | Swedish | Fristedt (2016) | 312 |  |  |  |  | Very good | SPBB total score: rho= 0.20; transportation rho =0.26; stair climbing: rho=0.10; transfers: rho=0.04 ; food shopping: rho =0.29; travel for pleasure: rho =0.20; community activities: rho=0.27 (7-) |
| LSA-M | Swedish | Kammerlind (2014) | 298 | Doubtful | Weighted kappa = 0.50 (0.31–0.70) (-) | Doubtful | % 81 agreement (+) |  |  |
| LSA-M | Finnish | Portegijis (2014) | Baseline: 848; longitudinal: 39 | Inadequate | ICC (95% CI) =0.65 (0.43-0.80) (-) | Adequate | 62 % agreement (+) |  |  |
| LSA-M | Spanish and Portuguese | Curcio (2013) | 300 (39 for reliability) | Inadequate | 7-10 days: ICC (95 % CI)= 0.63 (0.39–0.79) (-) |  |  |  |  |
| LSA-M | French | Auger (2009) | 40 | Inadequate | 7-10 days: ICC (95% CI) =0.81 (0.65-0.90) (+) |  |  |  |  |
| LSA-M | English | Baker (2003) | 306 | Inadequate | 2 weeks: ICC (95% CI) = 0.49 (0.40–0.57) (-) |  |  | Adequate | Physical performance measure:  Rho=0.194; ADL: rho=–0.077; IADLS : rho=–0.067; SF12PCS: rho=0.083; SF12 MCS: rho= 0.123; GDS: rho=–0.17; comorbid cond: rho=–0.005; self-reported health:  Rho=0.092 (8-) |
| **Total** | | | **1+; 4- Insufficient; inconsistent. ICC ranges form 0.49-0.81** | | | **2+ Sufficient 62-81% agreement** | | **15-; Insufficient.** | |

ADL: Activities of Daily Living; CI: Confidence Interval; FES-I: Falls Efficacy Scale International; FFABQ: Fear of Falling Avoidance Behaviour Questionnaire; GDS: Geriatric Depression Scale; ICC: Intraclass Correlation Coefficient; IADL: Instrumental Activities of Daily Living; LSA-M: Life-Space Assessment-Maximal; MAT-sf: Mobility Assessment Tool short form; MMSE: Mini Mental State Examination; OR: Odds Ratio; ROB: Risk of Bias; SF-12 MCS: Short Form Health Survey 12 items Mental Component Scale; SF-12 PCS: Short Form Health Survey 12 items Mental Component Scale; SPPB: Short Physical Performance Battery; SRM: Standardized Response Mean; TUG: Timed-Up and Go

Table. Psychometric Properties of the modified LSA maximum score (LSA-M) (n=2)

| **Measure** | **Language** | **Author (year)** | **Sample size** | **ROB** | **Reliability** | **ROB** | **Convergent validity** | **ROB** | **Responsiveness** |
| --- | --- | --- | --- | --- | --- | --- | --- | --- | --- |
| Modified LSA-M | German | Ullrich (2021) | 65 for convergent validity; 55 for reliability; 32 for responsiveness | Inadequate | Within 3 days: ICC (95% CI)= 0.642 (0.456-0.774) (-) | Adequate | With SPPB: rho=0.28*; FFABQ: rho=-0.19; Walking episodes: rho=0.17;Steps: rho-0.33**; mean outdoor walking duration: rho=0.32**; mean outdoor walking distance: rho=0.36**; outdoor walking episodes: rho=0.31*. (*p<0.05; **p<0.01) (1+,6-) | Doubtful | Post-intervention; 12 weeks: SRM= 0.48 (+) |
| Modified LSA-M | German | Ullrich (2019) | 117 for convergent validity; 102 for reliability; 53 for responsiveness | Inadequate | Within 2 days: ICC (95% CI) = 0.80 (0.71-0.86) (+) | Very good | With SPPB: rho= 0.05; Gait speed: rho=0.13; TUG: rho=-0.08; MMSE: rho=0.15; GDS: rho=-0.16; FES-I: rho=-0.12; FFABQ: rho=-0.15; Lying min: rho=-0.07; sitting min: rho=-0.06; standing min: rho=0.28**; walking min: rho=0.27**;walking episodes: rho=0.16; steps: rho=0.29**; being active outdoors: rho=0.30**; mean outdoor walking duration: rho=0.31**; mean outdoor walking distance: rho=0.34**; outdoor walking episodes: rho=0.31**; max distance from home: rho=0.32**(*=p<0.05;**=p<0.01) (18-) | Doubtful | Baseline and Post-intervention; 12 weeks: p=0.001 SRM=0.60 (+) |
| **Total** | | | **1+; 1- Sufficient; inconsistent. ICC ranges from 0.64-0.80** | | | **24-; 1+ Insufficient.** Correlations with performance-based measures: 0.05 (SPPB) – 0.28 (SPPB) | | **2+ Sufficient SRM ranges from 0.48-0.6** | |

Table. Psychometric Properties of the Life-Space Questionnaire (LSQ)

| **Measure** | **Language** | **Author (year)** | **Sample size** | **ROB** | **Reliability** | **ROB** | **Measurement error** | **ROB** | **Convergent validity** |
| --- | --- | --- | --- | --- | --- | --- | --- | --- | --- |
| LSQ | English | Stalvey (1999) | 242 (200 for reliability) | Inadequate | 1 year: weighted kappa=0.80 **(+)** | Inadequate | Items 1-5,8, and 9 had 90% test-retest agreement. Agreement on items 6 and 7 was 70%  And 73 %, respectively. **(+)** | Adequate | With visual acuity: rho=-0.10 (p=0.12); contrast sensitivity: rho=0.07 (p=0.25); useful field of view: rho=-0.24 (p=0.0001); mental status: rho=-0.29 (p=0.0001);depressive symp: rho=-0.17 (p=0.007); comorbidity index: rho=-0.08 (p=0.2); driving days/week: rho=0.08 (p=0.22); miles per week: rho=0.36 (0.0001); destinations: rho=0.19 (p=0.003); trips: rho=0.15 (p=0.02); POMA: rho=0.18 (p=0.01); ADVS: rho=0.14 (p=0.03). **(3+, 9-)** |
| **Total** | | | **1+ Sufficient** | | | **1+ Sufficient** | | **9-, 3+; Insufficient** | |

ADVS: Activities of Daily Vision Scale; LSQ: Life-Space Questionnaire; POMA: Performance Oriented Mobility Assessment; ROB: Risk of Bias

Table. Psychometric Properties of the modified Life-Space Questionnaire (LSQ)

| **Measure** | **Language** | **Author (year)** | **Sample size** | **ROB** | **Convergent validity** | |
| --- | --- | --- | --- | --- | --- | --- |
| Modified LSQ | English | Barnes et al. (2007) | 909 | Very good | With the ADL scale (rho = 0.23, p < .001), Rosow-Breslau scale (rho = 0.27, p < .001), and a measure of IADLs (rho = 0.32, p < .001). (3-) | |
| **Total** | | | **3-; Insufficient** | | |  |
