# Supplementary material for: Assessing the measurement properties of life-space mobility measures in community-dwelling older adults: a systematic review
Source: Age Ageing. 2023 Oct 30;52(Suppl 4):iv86–99. doi: 10.1093/ageing/afad119 (PMC10615067; doi:10.1093/ageing/afad119)

**Appendix F. Subgroup analysis of reliability values of the LSA-C**

**Figure. Subgroup analysis of reliability values of the LSA-C by risk of bias**


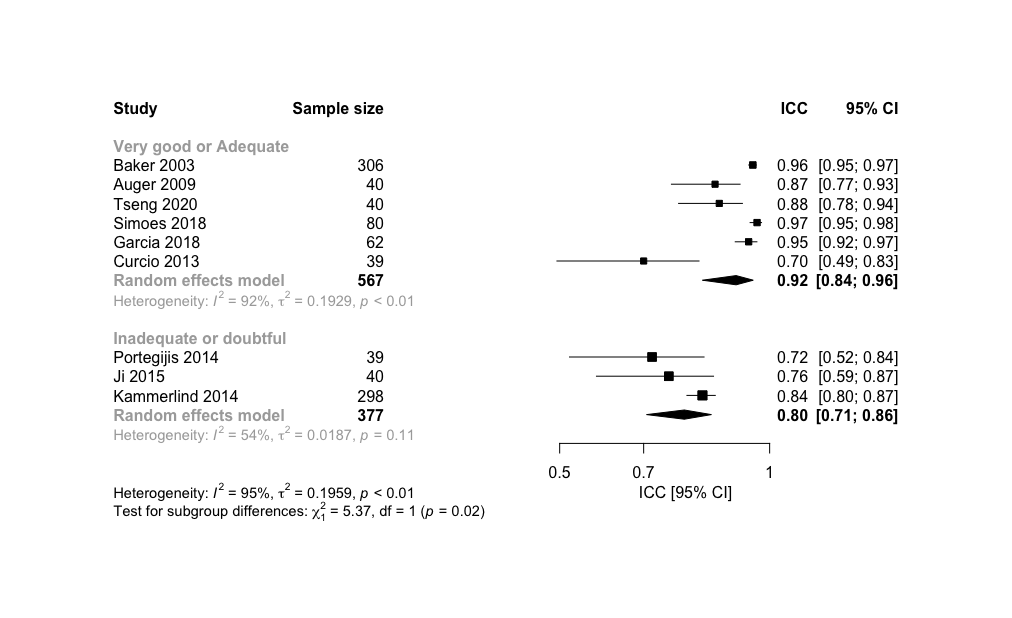


**Figure. Subgroup analysis of reliability values of the LSA-C by mean age**


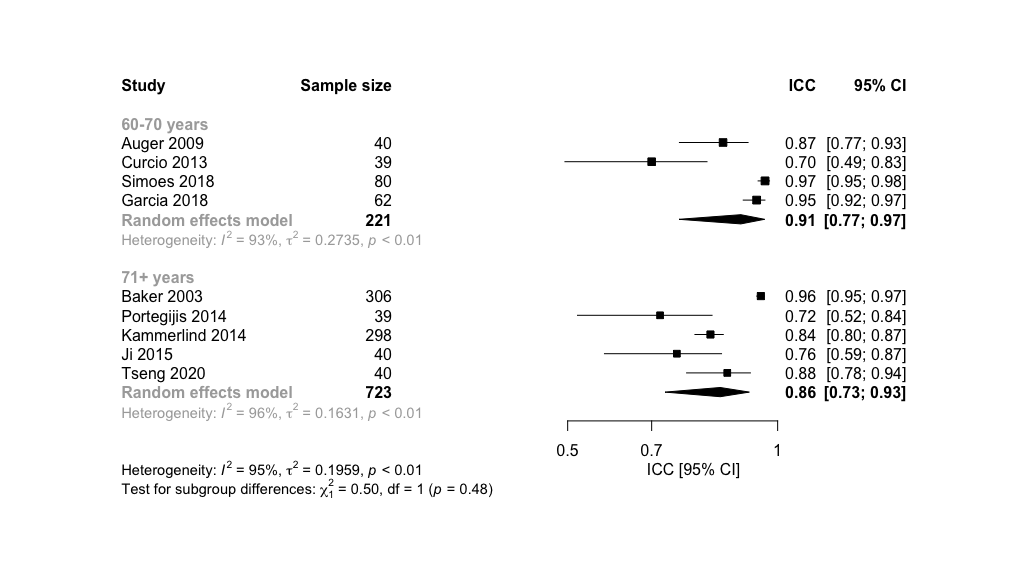

Supplement: aa-23-0362-File008_afad119 [file aa-23-0362-file008_afad119.docx]
