# Supplementary material for: Assessing the measurement properties of life-space mobility measures in community-dwelling older adults: a systematic review
Source: Age Ageing. 2023 Oct 30;52(Suppl 4):iv86–99. doi: 10.1093/ageing/afad119 (PMC10615067; doi:10.1093/ageing/afad119)

**Appendix H. Forest plots were performed for LSA-C against IADL and GDS**

**Figure. Forest plot of the LSA-C against measures of Instrumental Activities of Daily Living (IADL)**

**
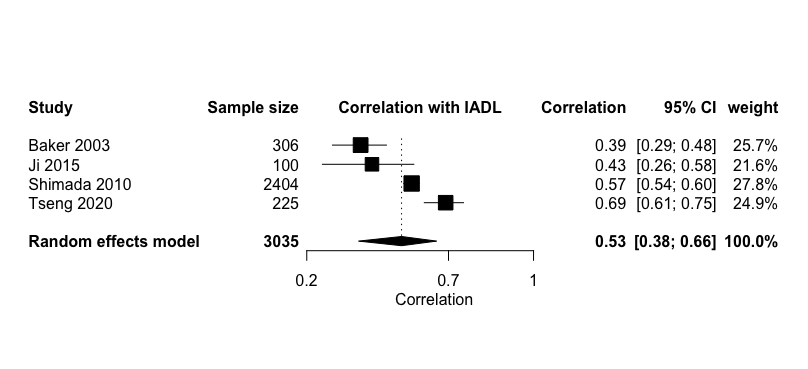
**

**Figure. Forest plot of the LSA-C against the Geriatric Depression Scale (GDS)**


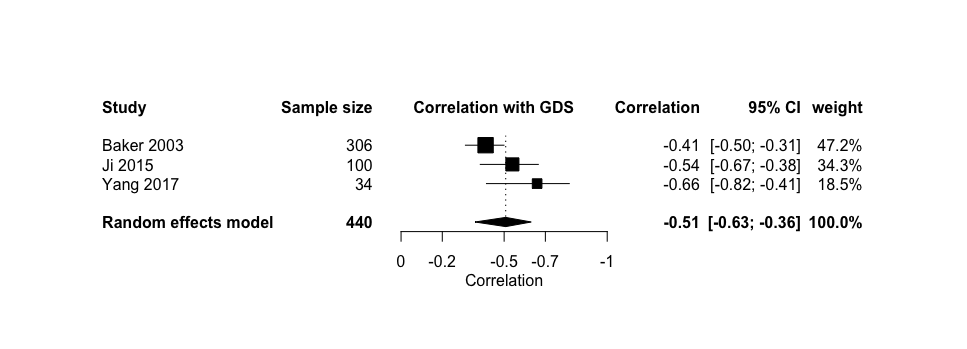

Supplement: aa-23-0362-File009_afad119 [file aa-23-0362-file009_afad119.docx]
